# Genome-wide identification, characterization and expression analysis of *populus* leucine-rich repeat receptor-like protein kinase genes

**DOI:** 10.1186/1471-2164-14-318

**Published:** 2013-05-10

**Authors:** Yanjun Zan, Yan Ji, Yu Zhang, Shaohui Yang, Yingjin Song, Jiehua Wang

**Affiliations:** 1 School of Environmental Science and Engineering, Tianjin University, Tianjin, 300072, China

**Keywords:** *Populus trichocarpa*, Leucine-rich repeat receptor-like kinase (LRR-RLK), Phylogenetic analysis, Motif elicitation, Expression profiling

## Abstract

**Background:**

Leucine-rich repeat receptor-like kinases (LRR-RLKs) comprise the largest group within the receptor-like kinase (RLK) superfamily in plants. This gene family plays critical and diverse roles in plant growth, development and stress response. Although the LRR-RLK families in *Arabidopsis* and rice have been previously analyzed, no comprehensive studies have been performed on this gene family in tree species.

**Results:**

In this work, 379 *LRR-RLK* genes were retrieved from the *Populus trichocarpa* genome and further grouped into 14 subfamilies based on their structural and sequence similarities. Approximately 82% (312 out of 379) of the *PtLRR-RLK* genes are located in segmental duplication blocks indicating the role of duplication process in the expansion of this gene family. The conservation and variation in motif composition and intron/exon arrangement among PtLRR-RLK subfamilies were analyzed to provide additional support for their phylogenetic relationship and more importantly to indicate the potential divergence in their functions. Expression profiling of *PtLRR-RLKs* showed that they were differentially expressed in different organs and tissues and some *PtLRR-RLKs* were specifically expressed in meristem tissues, which indicated their potential involvement in tissue development and differentiation. For most *AtLRR-RLKs* with defined functions, *Populus* homologues exhibiting similar expression patterns could be identified, which might indicate the functional conservation during evolution. Among 12 types of environmental cues analyzed by the genome-wide microarray data, *PtLRR-RLKs* showed specific responses to shoot organogenesis, wounding, low ammonium feeding, hypoxia and seasonal dormancy, but not to drought, re-watering after drought, flooding, AlCl_3_ treatment and bacteria or fungi treatments.

**Conclusions:**

This study provides the first comprehensive genomic analysis of the *Populus LRR-RLK* gene family. Segmental duplication contributes significantly to the expansion of this gene family. *Populus* and *Arabidopsis LRR-RLK* homologues not only share similar genetic structures but also exhibit comparable expression patterns which point to the possible functional conservation of these LRR-RLKs in two model systems. Transcriptome profiling provides the first insight into the functional divergence among *PtLRR-RLK* gene subfamilies and suggests that they might take important roles in growth and adaptation of tree species.

## Background

Plant cells are able to sense and transduce signals through cell surface receptors which mediate the cell-to-cell communication by binding to the extracellular ligands and possessing protein kinase catalytic activities [1]. In 1990, the first plant receptor-like kinase (RLK) was identified in maize [2] and since then, many RLKs have been identified from other plant species. According to the classification based on the extracellular domains, the major group of plant RLK is the leucine-rich repeat RLK family (LRR-RLK) [3]. The structural features of LRR-RLKs include an extracellular receptor domain to perceive signals, a single-pass transmembrane domain to anchor the protein within the membrane and a cytoplasmic serine/threonine (ser/thr) protein kinase domain to transduce the signal downstream *via* autophosphorylation followed by further phosphorylation of specific substrates [4,5].

Previous reports have classified plant *LRR-RLK* genes into two broad categories [3]. First, they are important in plant growth and development including morphogenesis, organogenesis and hormone signaling. Second, many LRR-RLKs respond to abiotic and biotic stress and therefore could be defense-related. Some LRR-RLKs have been demonstrated to possess dual functions due to the cross-talk between defense and developmental pathways or due to the recognition of multiple ligands by one signal receptor [6]. For instance, ERECTA is involved in both ovule development and resistance to bacterial wilt [7,8]. Although important progress has been made in understanding LRR-RLK functions in recent years, open questions still remain for most LRR-RLKs. The phenotypes associated with various *LRR-RLK* mutants show that they play roles in diverse processes during growth and development [9]. Meanwhile, the functional redundancy of *LRR-RLK* family members definitely adds to the complexity of the signaling network they mediate. For example, CLAVATA1 (CLV1) forms a receptor complex with CLV2 upon perception of the CLV3/ESR-related (CLE) peptide derived from CLV3 in the shoot apical meristem to regulate the expression pattern of the stem cell-promoting transcription factor WUSCHEL (WUS) [10-12]. In parallel with CLV1, additional receptors, namely Barely any Meristem (BAM1, BAM2, and BAM3), exhibit similar sequences as CLV1 but perform seemingly contradictory functions. While CLV1 promotes stem cell differentiation, BAM receptors are required for stem cell maintenance [13]. It has been shown that CLV1 and BAM receptors have retained significant similarity in their biochemical function and the differences in their genetic functions appear to be largely driven by their distinctive expression patterns [13].

LRR-RLKs seem to have evolved to acquire novel and diverse functions through neofunctionalization and subfunctionalization by extensive gene duplication [14]. The drastic expansion of this gene family in the land plant lineage is regarded as a plant-specific adaptation for extracellular signal sensing and propagation [15,16]. As a forest model organism, poplar is a fast-growing diploid plant that has attracted much attention since its whole genome being sequenced [17]. The structural features and expression profiles of *LRR-RLK* gene family members have been extensively described in *Arabidopsis* and rice, however, there has been much less information about this family in woody species including poplar. In the current study, the entire *LRR-RLK* gene family of *Populus trichocarpa* was comprehensively identified and analyzed by incorporating sequence phylogeny, gene organization, conserved motif, expression profiling, and gene adaption analysis. Our results provide a framework for further functional investigation on *Populus LRR-RLKs* and contribute to a better understanding of the complexity of *LRR-RLK* gene family in higher plants.

## Results and discussion

### Composition and phylogenetic analysis of *LRR-RLK* gene family in *populus trichocarpa*

**Figure 1:**
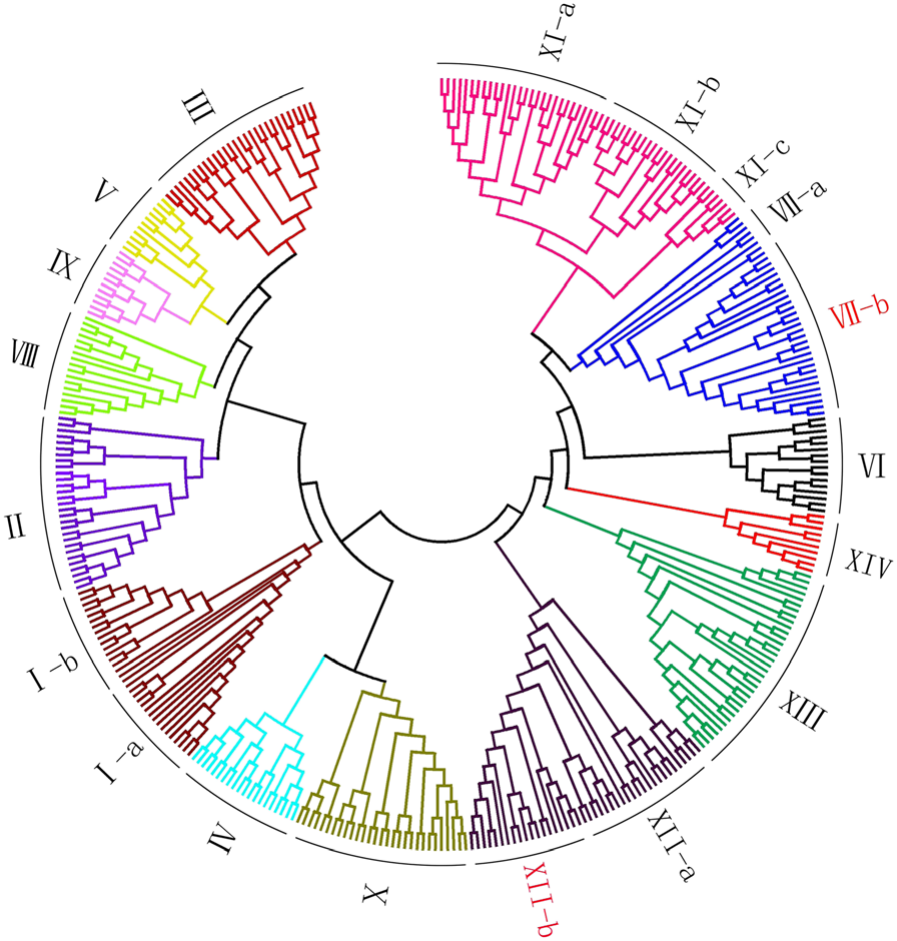
**Unrooted phylogenetic tree of LRR-RLK genes retrieved from *Populus trichocarpa*.** The phylogenetic tree was established with amino acid sequences of the LRR domains by the Neighbor-Joining (NJ) method. All PtLRR-RLKs were divided into fourteen distinct subfamilies (I to XIV).

To date, approximately 213 and 309 *LRR-RLK* genes have been identified in the fully sequenced *Arabidopsis* and rice genomes, respectively [18,19]. In this work, a larger *LRR-RLK* gene family composed of 379 members was identified in the *P. trichocarpa* genome. The number of LRR-RLK genes in *Populus* is roughly 1.78 fold of that in *Arabidopsis*, which is consistent with the ratio of putative *Populus* homologues to each *Arabidopsis* gene (1.4~1.6) [17]. The detailed information of *LRR-RLK* family genes in *Populus* including the accession numbers and the characteristics of the encoded proteins is listed in Additional file 1 and the summarized information concerning each group or subgroup is presented in Table 1. Since the diversity of extracellular domains (ECDs) represents the capability of LRR-RLKs to recognize various ligands and thus constitute the basis of their functional versatility [20], we first identified the ECD for each PtLRR-RLK and constructed the phylogenetic tree to determine their evolutionary relationship (Figure 1, Additional file 2). It has been shown that many events which resulted in the fusion between ECDs and kinase domains occurred early in land plant evolution, thus *RLK* genes with related kinase sequences tend to have similar ECDs [15,20,21]. In this work, the phylogenetic relationship among the PtLRR-RLKs was also examined based on their catalytic kinase domains and similar categories were obtained (Additional file 3). Since the nodes of the phylogenetic tree based on the ECDs exhibit the best confidence of support, PtLRR-RLKs were classified into 14 subfamilies (I to XIV) accordingly (Figure 1). No well-supported positions could be identified for six PtLRR-RLKs, so they were not included in the phylogeny (Additional file 1). When PtLRR-RLKs were clustered with AtLRR-RLKs (Additional file 4), the numbering for the *Populus* LRR-RLK subfamilies was determined based on the nomenclature of the majority of *Arabidopsis* homologues within the same group. The *Populus* subfamilies I, II, III and XIII were grouped together with *Arabidopsis* LRR-RLKs involved in organ/tissue development and with the ones involved in defense signaling. Group IV included only two *Arabidopsis* Inflorescence Meristem Receptor-like Kinase (*IMK*) genes which are involved in cell fate specification and proliferation. Group V included the *Arabidopsis* Strubbelig-receptor Family (*SRF*) gene family members that affect different aspects of cell wall biology [22,23] and the *SCM* gene involved in root hair specification [24]. Group VI, VII, VIII and IX had no *Arabidopsis* orthologs with identified functions. Group X was grouped together with *Arabidopsis* genes involved in brassinosternoid and peptide signaling such as BAK1-interacting Receptor1 (*BIR1*), BAK1-interacting Receptor-like (*BIR-like*), and Phytosulfokin receptor1-2 (*PSKR1-2*) genes [25]. Subgroups XI-a and XI-b were represented with *Arabidopsis LRR-RLKs* with important roles in organ morphogenesis, cell fate specification and vascular development such as *CLV1* and Phloem Intercalated with Xylem (*PXY*) [26-29], while in the subgroup XI-c, PEP1 receptor1-2 (*AtPEPR1-2*) is involved in abscisic acid signaling and defense response [30,31]. For group XII, subgroup XII-b is *Populus* specific and subgroup XII-a was clustered with Flagellin-sensitive2 (*FLS2*) and EF-Tu receptor (*EFR*) which take part in innate immunity against pathogens [32,33]. Group XIV only included Excess Microsporocytes1 (*EMS1*) gene, which is involved in endosperm and pollen development [34]. The dispersal pattern of 63 *Arabidopsis* LRR-RLKs with well-defined roles prompted consideration of the ancestral role of distinct PtLRR-RLK subfamilies and there is a possibility that *PtLRR-RLKs* belonging to distinct subfamilies perform certain functions in different developmental aspects. For example, the subgroups XI-a and b are more likely to be involved in plant growth and development, while XI-c could be more likely to take roles in plant-microbe interactions. The large size of the *Populus* LRR-RLK gene family has been regarded as a indication of a great need for LRR-RLK genes to participate in more complicated transcriptional regulations in woody species [20]. Meanwhile, the species-specific genes could play important roles in plant responses to a variation of biotic factors, such as the variation of the spectrum of pathogens [35], so it would be very attractive to investigate the functions of the poplar-specific subgroups identified in this work.

**Table 1:** **Group and subgroup designations of LRR-RLK proteins from *Populus trichocarpa***

**Groups**	**Subgroups**	**Gene number**	**PI**	**MW(kDa)**	**aa length**	**With signal peptide**	**Homologous *Arabidopsis *genes based on LRR**
I	a	16	5.29-7.94	43.32-104.52	391-934	56.3%	FRK1, IOS1, MEE39
	b	17	5.24-8.86	49.58-101.98	455-923	58.8%	FEI1-2, TMKL1
II		29	5.19-9.45	68.92-79.48	564-715		NIK1, NIK2, SARK,NIK3, SERK1-5, MRH1
III		29	5.85-9.19	59.07-74.62	547-684	65.5%	RLK902, RKL1, RUL1, PRK2A
IV		20	5.43-8.66	59.51-132.35	546-1211		IMK2, IMK3
V		11	5.82-8.03	67.75-86.62	625-794	54.6%	SRF1-8, SCM
VI		16	5.68-9.21	74.95-114.81	673-1072		
VII	a	6	5.22-8.72	74.08-133.71	673-1221	83.3%	
	b	30	6.11-8.67	66.14-128.21	599-1162	30.0%	***Populus*-specific**
VIII		17	5.29-8.16	48.97-106.77	449-978	58.8%	
IX		12	5.31-6.68	98.97-103.46	908-949	100%	
X		29	5.22-8.77	49.72-130.50	446-1205	82.8%	BIR1, PSKR2, PSKR1,BRL2, BRI1, BRL1,BRL3
XI	a	28	5.15-8.03	77.44-113.17	701-1026	85.7%	HAESA, XIP1, HAIKU2, RLK7, PXY, BAM1-3, CLV1, MOL1
	b	20	5.33-6.06	96.55-136.72	870-1254	55.0%	GSO1-2, EDA23, ERL1-2, ERECTA
	c	7	5.94-8.68	96.63-127.25	887-1145	100.0%	PEPR1-2
XII	a	23	5.22-8.56	85.98-126.86	783-1158	60.9%	FLS2, EFR
	b	19	5.61-8.44	53.24-115.95	489-1067	52.6%	***Populus*-specific**
XIII		34	5.00-9.14	62.81-135.28	565-1605	47.1%	MEE62, TOAD1-2
XIV		10	5.42-8.95	63.75-134.48	574-1237	30.0%	EMS1

Sequence characteristics of each *PtLRR-RLK* gene subfamily and its phylogenetic relationship with *Arabidopsis* LRR-RLKs with known functions are shown. Six PtLRR-RLK proteins did not fit well into clusters. Subgroups that do not include any *Arabidopsis* LRR-RLK proteins are described as *Populus*-specific. Information on the AGI code, gene full-name and abbreviation for each *AtLRR-RLK* gene with defined functions is presented in the Additional file 18.

### Intron–exon organizations of *PtLRR-RLKs*

**Figure 2:**
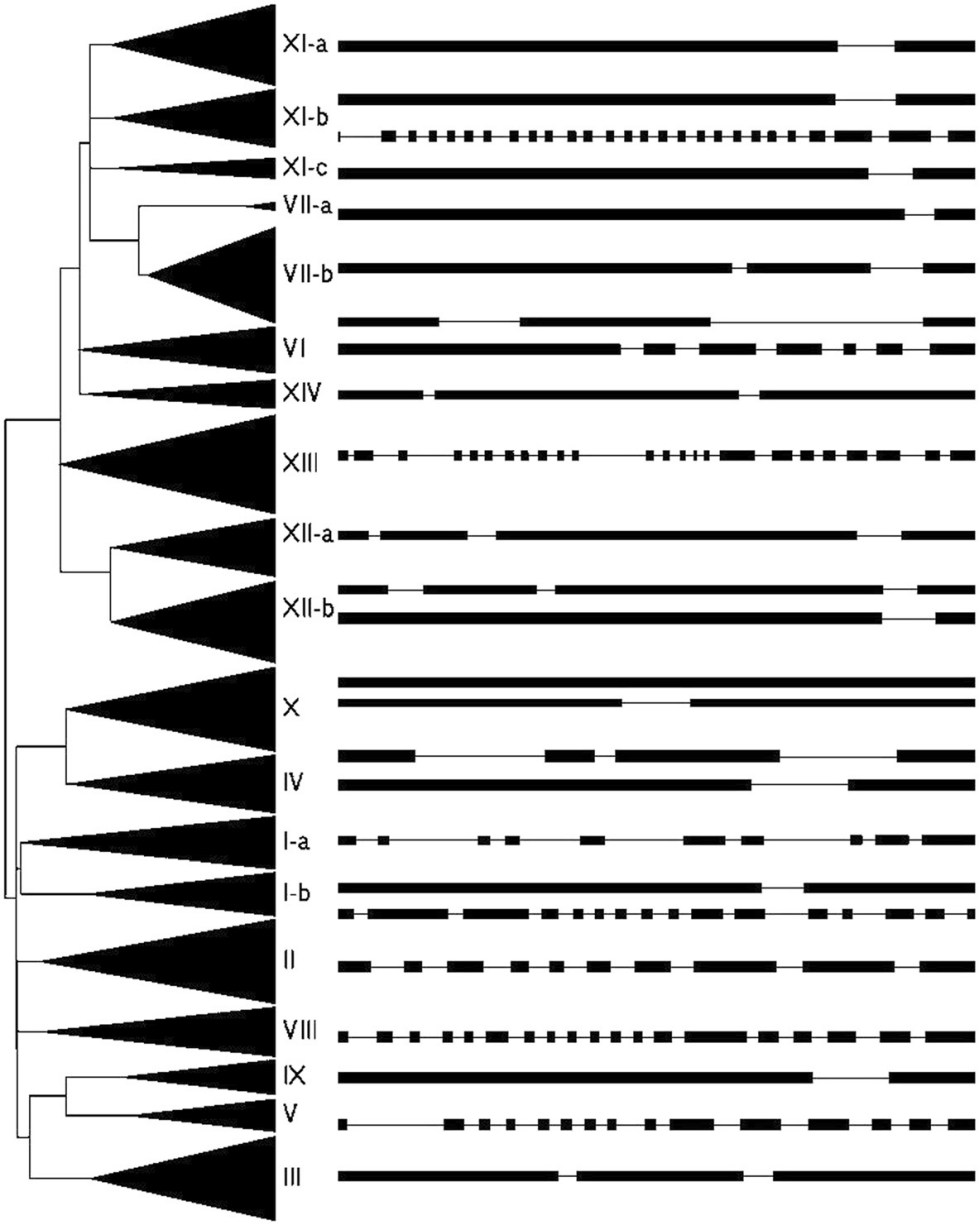
**Representative intron-exon structure of each LRR-RLK gene subfamily in *Populus*.** Exons and introns are represented by boxes and lines, respectively; and were drawn to scale with the full encoding regions of their respective gene.

The presence of multiple introns has been shown to be essential for ERECTA expression in *Arabidopsis*[36], so the intron–exon organizations of PtLRR-RLKs were examined for a clearer understanding of their potential functions. Additional file 5 provides the detailed illustration of the distribution and position of introns for each *PtLRR-RLK* genes and Figure 2 listed the representative intron/exon structures and their distributions among different gene subfamilies (Figure 2). Out of 379 *Populus LRR-RLK* genes, 30 had alternative mRNA splicing modes and 25 genes had no intron. One, two, three, four, and five introns was found in 153, 54, 23, 6 and 3 genes, respectively. One hundred and fifteen genes had more than five introns and 72 out of them had more than ten introns (Additional file 5). In terms of exon/intron organization, most of the closely related *Populus LRR-RLK* genes have roughly the same number and location of introns (Figure 2), which strongly supports their close evolutionary relationship. *Populus* and *Arabidopsis* genes belonging to the same subfamilies also exhibit similar genomic features. For example, the gene structure of the *Populus* subfamily XI were fairly simple and has only one or two introns over their full length sequence, except three genes in the subgroup XI-b which contain as many as 26 introns. *Arabidopsis* homologues of this group have been shown to play important roles in plant development and organogenesis and most of them contained less than two introns except ERECTA and ERECTA-LIKE1-2 (ERL1-2) which contained as many as 26 introns, this is the most complicated intron/exon structure of *AtLRR-RLKs*. Although all of the 63 AtLRR-RLKs with known functions could be matched with *Populus* homologues with similar intron/exon structures, the exactly same genetic structures as AtLRR-RLKs were only found in *Populus* group V, XI and XIII and interestingly, all of them are developmental genes responsible for cell fate specification and morphogenesis (Additional file 6). These results confirmed that the common ancestral genes of *PtLRR-RLKs* and *AtLRR-RLKs* already possess multiple intron/exon structures and probably the complicated mRNA processing modes as well. Meanwhile, it seemed possible that the development-related LRR-RLK genes are more conserved in the evolution of genetic structures than the defense-related LRR-RLKs due to their indispensible roles for plant life.

### Conserved motifs of *PtLRR-RLK* genes

To further reveal the diversification and functional potentials of *Populus* LRR-RLKs, their conserved motifs were investigated and the consistency of domain arrangement for each subfamily was determined using the Multiple EM for Motif Elicitation (MEME) motif detection software [37]. The LRR motif is usually composed of 20–29 residues with conserved leucines [38] and the consensus residues within the LRR motif were thought to provide a structural skeleton for protein-protein interactions and non-consensus residues within LRRs are though to determine the specificity of such interactions [39]. In total, 17 LRR-related motifs were identified among PtLRR-RLK family members and the basic LRR motif was concluded as LxxLxLxxNx L/f sGx I/l Pxx l/I gxLxx, which shows a good match to the plant LRR consensus LxxLxLxxNxLxGxIPxxLxxLxx and was slightly different from the basic LRR motif in rice (LxxLxLxxNx L/f xGx I/l Pxx l/i Gx L/c xx) [19]. The most conserved amino acid residues in *Populus* LRR motifs were Gly at position 1, Pro at position 4, Leu at position 13, 16 and 18, and Asn at position 21. Ile at positions 3 and Leu at position 7 are often substituted by each other and Leu at position 23 is often replaced by Phe (Table 2). Some repeats contain additional conserved residues in other positions, such as a Gly at position 8 of the M23 repeat; Ser residue at position 19 of the M1, M5, M7, M9, M12 and M19 repeat. Since the repetitive structure of LRR makes it capable of the rapid generation of new variants by duplications and deletions of entire repeats [40], the repeat number and distribution of LRR motif have been regarded as important parameters to reflect the evolutionary history. For PtLRR-RLKs, most of the closely related members in the phylogenetic tree kept similar motifs, providing additional support for their phylogenetic relations (Additional file 7). The conserved motifs in the LRR-RLK proteins within the same subfamily may suggest their functional similarities and divergence in motif composition may indicate their functional diversity [35]. Although no group- or subgroup-specific LRR motif was identified, members of different subfamilies did exhibit various degree of complexity in terms of the LRR motif composition (Additional file 7). The most complicated motif composition was observed for the group XI which included all 17 types of LRR motif and in contrast, the group I and II had only 3 to 7 LRR motifs. In addition to the motif composition, the similarity in terms of the arrangement of different LRR motifs also varied among subfamilies (Additional file 7). The arrangement was almost identical for members of subfamily II, III, V, IX and XII-b. The variation in LRR patterns gets more obvious among the members of other subfamilies, although after careful comparison, several clades sharing a regular motif arrangement could still be identified for each subfamily (Additional file 7). The high divergence in the alignments of LRR motif within one subfamily could reflect the functional diversity among their members. In addition to LRR motifs, non-LRR motifs were also identified in the extracellular regions of PtLRR-RLK (Additional file 8). Common motifs including M4, M14 and M17 could be identified in the N-terminal for most of PtLRR-RLKs, while M13 could be found at the C-terminal for most PtLRR-RLKs. Different from these common motifs are certain non-LRR motifs which appear to be subfamily-specific, for example, the motif M24 only appeared in most members of VIII (Additional file 8). PtLRR-RLKs sharing the same or similar motif composition and arrangement could be identified for 50 out of 63 AtLRR-RLKs with known functions (Table 3 and Additional file 9), which supports the theory that the domain organization of most RLK/Pelle subfamilies was established before the monocot–dicot split [16].

**Table 2:** **Major motifs in the predicted LRR domains of *Populus *LRR-RLKs**

**LRR motifs**	**1**	**2**	**3**	**4**	**5**	**6**	**7**	**8**	**9**	**10**	**11**	**12**	**13**	**14**	**15**	**16**	**17**	**18**	**19**	**20**	**21**	**22**	**23**	**24**
M1	**G**	x	**I**	**P**	x	x	L/i	G	x	L	x	x	**L**	x	x	**L**	D	**L**	S	x	**N**	x	L/f	s/t
M2	**G**	x	**I**	**P**	x	x	l/i	G	N	l	t	x	**L**	x	x	**L**	x	**L**	x	x	**N**	x	L/f	x
M3	**G**	x	x	p	x	x	l	x	x	l	x	x	**L**	x	x	**L**	x	**L**	s	x	**N**	x	F/l	s/t
M5	**G**	x	I/l	**P**	x	x	l	g	x	L	x	x	**L**	x	x	**L**	D	**L**	S	x	**N**	x	**L**	t
M6	**G**	x	I	**P**	x	x	L/i	x	x	C	x	x	**L**	x	x	L	x	L	x	x	**N**	x	L/f	s
M7	**G**	p	I/l/v	**P**	x	x	l	x	x	l	x	x	**L**	x	x	L	D	L	S	x	**N**	x	L/f	s
M8	**G**	x	**I**	**P**	x	e	l/i	G	x	**L**	x	x	**L**	x	x	**L**	x	**L**	x	x	**N**	x	**L**	x
M9	**G**	x	I/l	**P**	x	x	l/f/i	g	n	L	s	n	**L**	x	x	**L**	d	**L**	S	x	**N**	x	L/f	x
M10	**G**	x	I/l	**P**	x	x	l/f/i	g	N	L	x	n	**L**	x	x	L	x	**L**	s	x	**N**	x	L/f	s
M11	**G**	x	i	p	x	s	l	g	x	l	x	n	**L**	x	x	**L**	x	**L**	s	x	**N**	x	L/f	s
M12	G	x	x	p	x	x	l/f/i	x	x	l	x	n	**L**	x	y	L	d	**L**	S	x	**N**	x	l/F	x
M15	**G**	x	i	P	x	x	l	x	x	l	x	x	**L**	x	x	L	d	**L**	s	x	**N**	x	L/f	s
M16	**G**	x	I/l	P	x	x	l/i	g	x	L	x	x	**L**	x	x	**L**	x	L	s	x	**N**	x	L/f	x
M18	**G**	s	i/l/f	**P**	x	x	L/i	g	N	L	x	x	**L**	x	x	L	x	L	x	x	**N**	x	L/f	x
M19	**G**	x	I/l	**P**	x	s/e/a	l/f	x	x	l	x	x	**L**	x	x	L	d	L	S	x	**N**	x	**L**/f	s
M20	**G**	x	i/l	P	x	x	l/i	x	x	l	x	n	**L**	x	x	L	x	L	x	x	**N**	x	l/F	s
M23	**G**	x	I/l	**P**	x	e	L/i	**G**	n	**L**	x	x	**L**	x	x	L	x	l	x	x	**N**	x	L/f	s/t

If the bits value of the amino acid at this position is smaller than 0.5, it is represented with x; 1>bits ≥ 0.5, with lowercase; 2> Bits ≥ 1, with capital letter; 3> bits ≥ 2, with bold capital; bits≥3, with underlined capital letter in bold.

**Table 3:** ***Arabidopsis *and *Poplus LRR-RLK *genes resembling each other in motif configuration**

**At Gene Family**	**Gene name**	**At Gene locus**	**Pt Gene locus**
II			POPTR_0004s10790.1
	NIK1	At5G16000	POPTR_0017s14360.1
	NIK2	At3G25560	POPTR_0008s10970.1
	NIK3	At1G60800	POPTR_0010s14410.1
			POPTR_0008s19310.1
	SARK	At4G30520	POPTR_0006s19320.1
			POPTR_0018s11030.1
	SERK3	At4G33430	POPTR_0001s21420.1
	SERK4	At2G13790	POPTR_0003s01740.1
	SERK5	At2G13800	
	SERK1	At1G71830	POPTR_0005s08500.1
	SERK2	At1G34210	POPTR_0013s12150.1
			POPTR_0019s11740.1
III	PRK2A	At2G07040	POPTR_0018s14390.1
			POPTR_0006s07820.1
	RUL1	At5G05160.1	POPTR_0019s15210.1
			POPTR_0013s15480.1
	RLK902	At3G17840	POPTR_0012s04170.1
	RKL1	At1G48480	POPTR_0015s04920.1
	LRR1	At5G16590	
	TMKL1	At3G24660	POPTR_0002s25300.1
V			POPTR_0001s16170.1
	SRF7	AT3G14350	POPTR_0003s07130.1
	SRF6	AT1G53730	POPTR_0004s00710.1
	SRF8	AT4G22130	POPTR_0011s01540.1
	SRF4	AT3G13065	POPTR_0007s15170.1
	SRF5	AT1G78980	POPTR_0014s00340.1
	SRF2	AT5G06820	POPTR_0006s20510.1
	SCM	AT1G11130	POPTR_0004s03720.1
	SRF3	AT4G03390	POPTR_0011s04540.1
	SRF1	AT2G20850	POPTR_0019s13490.1
			POPTR_0013s14080.1
VI-b	MRH1	AT4G18640	POPTR_0011s07250.1
			POPTR_0004s05680.1
X-a			POPTR_0008s07830.1
			POPTR_0002s24100.1
	BIR1	AT5G48380	POPTR_0017s03710.1
			POPTR_0010s18540.1
			POPTR_0010s18550.1
X-b	BRI1	At4G39400	POPTR_0007s06940.1
	BRL1	At1G55610	POPTR_0001s47680.1
	BRL3	At3G13380	POPTR_0011s17240.1
	PSKR1	AT2G02220	POPTR_0008s14390.1
			POPTR_0010s10790.1
	PSKR2	AT5G53890	POPTR_0011s11780.1
XI	HAE	At4G28490	POPTR_0007s01340.1
			POPTR_0017s04790.1
	XIP1	At5G49660	POPTR_0002s11230.1
	RLK7	At1G09970	POPTR_0002s10700.1
	HAIKU2	At3G19700	POPTR_0009s08540.1
	MOL1	At5G51350	POPTR_0001s00820.1
	PXY	At5G61480	POPTR_0003s10680.1
	CLV1	At1G75820	POPTR_0002s02140.1
			POPTR_0005s26300.1
	BAM1	At5G65700	POPTR_0007s14500.1
	BAM2	At3G49670	
	BAM3	At4G20270	POPTR_0001s12420.1
			POPTR_0003s15600.1
	GSO1	At4G20140	POPTR_0001s12290.1
	EDA23	At5G44700	
	PEPR2	At1G17750	POPTR_0008s00810.1
	PEPR1	At1G73080	POPTR_0008s00970.1
XIII-a	FEI1	AT1G31420	POPTR_0001s01120.1
	FEI1	AT2G35620	POPTR_0012s07290.1
XIII-b	ERL1	At5G62230	POPTR_0015s14270.1
	ERL2	At5G07180	POPTR_0012s14250.1
	ERECTA	At2G26330	POPTR_0006s23680.1

When the trans-membrane (TM) domains were predicted by TMHMM, in a total of 379 PtLRR-RLKs, 339 had one TM and 26 PtLRR-RLKs did not have any TM. Further analysis of the remaining 14 PtLRR-RLKs with two TMs revealed one of them is atypical. The RLK domain of most PtLRR-RLK consists of approximately 250–280 amino acid residues with a maximum of 324 and a minimum of 168. In literature, plant RLK could be divided into 12 conserved subdomains (I–XII) from N- to C-terminal [41]. In the 2- lobed structure of the RLK domain, the smaller lobe is composed of subdomains I to IV and is involved in anchoring and orienting the nucleotide. The larger lobe is composed of subdomain VI to XI and is largely responsible for binding the peptide substrate and initiating phosphor-transfer [41]. In the kinase part of all PtLRR-RLKs, 25 motifs are identified which are similar to those identified for rice LRR-RLKs and were named as 1 to 25 according to the frequencies of their appearance (Table 4). Although most motifs did not seem to be subfamily-specific, motif 10, 13 and 16 only appeared in the subgroup VII and motif 15, 24 and 25 only showed up in the subfamily XII. Since only these two subfamilies included a *Populus*-specific clade in the phylogenic tree, these specific motifs may, to some extent, attribute to the functional divergence of these subfamily members in poplar.

**Table 4:** **Major motifs in the predicted protein kinase domains of *Populus *LRR-RLKs**

**Motif**	**Sequence**	**Corresponding motif sequence in rice RLK**
20	**F**N/DSN/K**YC**	
6	I/l**G**x**G**Gf**G**x**VYK/**rA/GxL/mx	xxNlI/L**G**x**G**gf**G**x**VY**KG/axLxxG
9	d**G**xxV**AVK**K/rL	x**VAV**/i**K**vLxxxx
5	ke**F**xx**E**v/ixx**L**gxi/lR**H**R**N**L/I**V**k**L**	xs**F**xx**E**c/vex**L**/isxv/iR**H**R**NL**/iVxL/ixG/txCxxxd
11	y**GFC**sxxkxxF	
23	**E**A/dS/TxQ/nL**S**xNQ/pS/T**SS**I/V**G**	
19	R**AY**/f**Y**y/wskD**EKL**	
15	**TA**/s**CS**S/GV/I**D**F/yQ/k**GNDFKA**	xx**G**nD/e**FKA**
4	**LVY**EY/FM/YxN**GSL**xxx**L**	**LVYE**Y/fMpN**G**S**L**xxx**L**Hxx
14	xexxxxx**LDW**	
3	x**R**lk/n**I**/vAxG/dV/a**A**xG/A**L**x**YLH**	LdW/lxx**R**lxx**I**AlG/Dvv**A**xG/Ax**YLH**
1	p**I**I/V**H**R**D**L/I**K**S/ps/n**NI**/V**LLD**xd/exeA/ph/kV/i/ls/a**DFG**L	xI/vv/i**H**R/c**D**I/l/v**K**s/pS**N**I/V**LLD**
18	**L**xxxxd**T/**S**H**V/IT/S**T**	
13	**LLMPDSSNWTS**/a	
25	**E**A/dS/Txq/n/hl/h**S**x**N**Q/pS/T**SS**I/V**G**	xssxsGSt/sxxefsxqxExxP
7	xA**GT**/sx**GY**x**APEY**axT	xxxxxxxx**GT**/si**GY**i**APEY**g/axx
22	xxxxxxxxxg**Y**r/k**A/**s**PE**xxxxkK/r	xxxxxx**GY**R**APE**vxxxxk/rS/tx
2	e**K**x**DVYSF/**y**GV**/i**V/**l**LL**/m**E**Ll**T/**s**G**K/rxP	Ks/g**DVY**/f**SF**/y**G**V/iV/l**LLE**ll**TG**K/RxPx
12	xxxd**L**ivx**WV/**a	x**L**vx**W**V/axxxxxxxxx
16	**GDL/**F**IS**S/A**L**L/M/sSS/P**A**/G**SS/**T**SS**	
17	xxxEv/iv/i**D**PxL/i	xev/iv/l**D**pxLxxx
21	L/F**H**T/G**AIDK**S/P**L**I/p/l**G**Q/N**GF/**y**D**H/A**E**I/Ln/i	
10	P/si/sg/aqh/nT/aL**LKDVLDQ/**p**R**L/IP/SP/L**P**E/kn/ke/ga/l/v**A**E/d**GV**	
24	xxsh/fg/egNSI/es/exn/e**K**/RV/I**ECL**V/IS**I**	
8	lki/v/lA/glx**C**t/vxxx**P**xx**RP**t**M**xe**V/**i	lxxvlxl/v/iA/gL**xG**txxx**P**xx**RP**x**M**xe

If the bits value of the amino acid at this position is smaller than 0.5, it is represented with x; 1>bits≥0.5, with lowercase; 2>bits≥1, with capital letter; 3>bits≥2, with bold capital; bits>3, with underlined capital letter in bold.

Kinase is commonly referred to as arginine-aspartate (RD) kinases if it is strongly activated by the phosphorylation of the activation loop and they usually contain an Arg(R) in the subdomain preceding the catalytic loop [41]. Conversely, a smaller number of kinases are referred to as Non-arginine-aspartate (non-RD) kinases which lack the conserved R in subdomain VI [42,43]. It has been proposed that the signal of pathogen recognition mediated by RLKs is usually through a non-RD kinase [44]. In PtLRR-RLKs, about half are RD-kinases including all the members of subfamily VII and IX. Interestingly, no *Arabidopsis* homolog with known functions has been identified for these two subfamilies and the VII-b subfamily is *Populus*-specific. In contrast, all members in the subfamily III, IV, V and XII are non-RD kinases although the *Arabidopsis* LRR-RLKs grouped with them take part in both defense and development (Table 1).

### Contributions of tandem and large-scale duplications to the family size of PtLRR-RLKs

**Figure 3:**
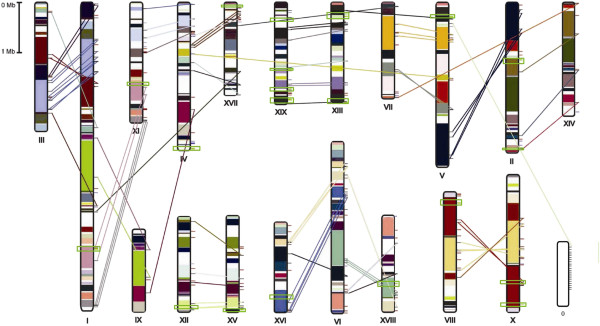
**Distribution of *LRR-RLK *genes on *Populus *chromosomes.** Genes are mapped to LG according the Joint Genome Institute Poplar Genome version 2.0. The schematic diagram of *Populus* chromosome organization arisen from the salicoid genome duplication event was adapted from Tuskan et al., (2006) [17]. Scale represents a 1 Mb chromosomal distance. Segmental duplicated homologous blocks are indicated with the same color and corresponding sister gene pairs were connected by colored lines. Tandemly duplicated genes are encompassed in the green boxes.

The explosion of members of a gene family has generally occurred as the result of repetitive tandem duplication (TD) and segmental and/or whole genome duplication events (S/WGD). PtLRR-RLK genes were comprehensively distributed within the poplar genome and 22 genes are localized to unassembled genomic sequence scaffolds and thus could not be mapped to any particular chromosome (Figure 3). Approximately 82% (312 out of 379) *PtLRR-RLK* genes are located in the replicated region, which is different from rice and *Arabidopsis* in which the frequencies of genes generated by S/WGDs are much lower (11% in rice and 26% in *Arabidopsis*) [45]. Among them, 140 genes lacked duplicates on the corresponding duplicated blocks, suggesting that dynamic rearrangement, mutation or segmental loss may have occurred following the segmental duplication. According to previous literature, a chromosome region containing two or more genes within 200 kb can be defined as a gene cluster [46]. In poplar, 72 *PtLRR-RLK* genes were located in 20 tandem duplication clusters (Figure 3). The smallest tandem duplication clusters consisted of only 2 genes and the largest cluster had 8 tightly linked genes on chromosome 15 and 19. The clusters were distributed unevenly among the 14 phylogenetic groups, and *Populus*-specific subgroup VII-b contains 6 clusters incorporating 86.7% of the genes of this subgroup. By contrast, group II, III, IV, V, VI, IX had no clusters present (Additional file 10).

### Differential expression profiles of *populus LRR-RLK* genes

**Figure 4:**
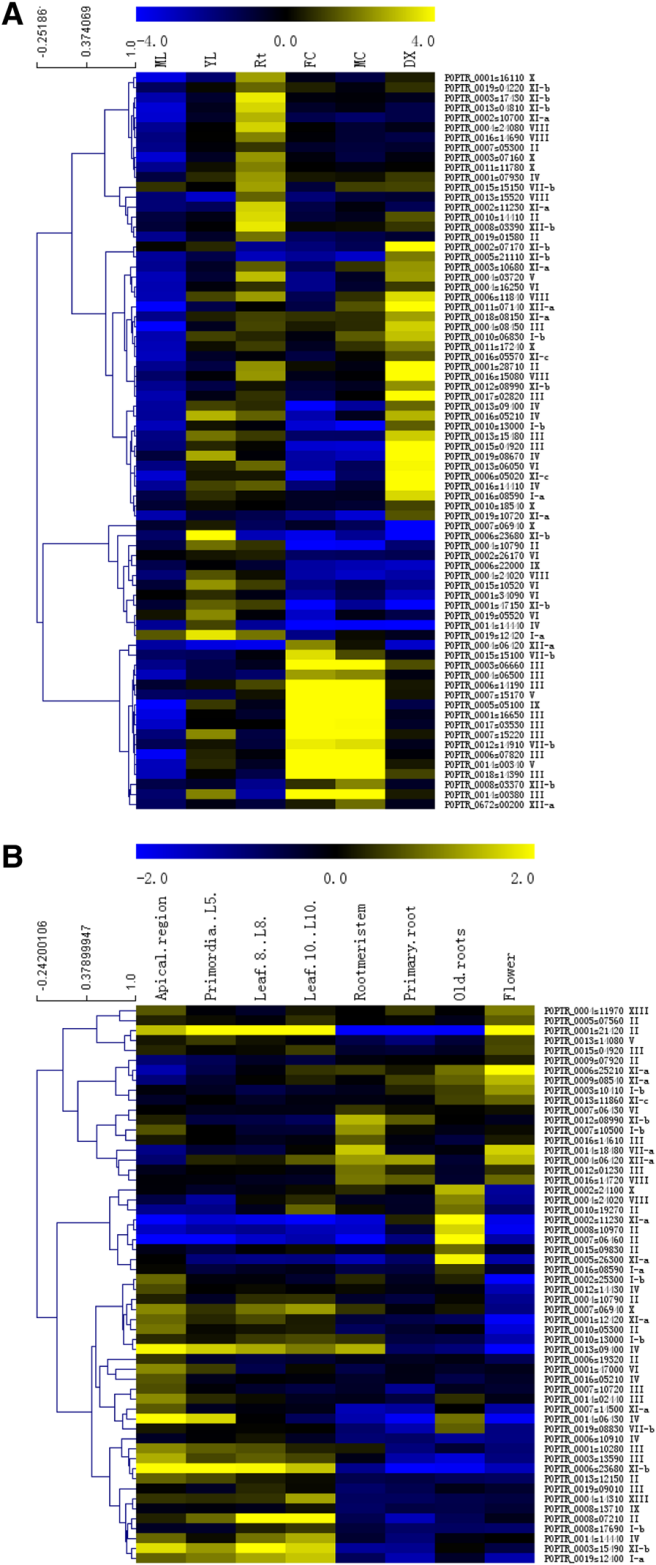
**The relative transcript accumulation of *Populus *LRR-RLK genes across different tissues.** The microarray-based expression data were downloaded from the Poplar eFP browser, gene-wise normalized and hierarchical clustered based on Pearson correlation. Color scale at the top of each dendrogram represents log2 expression values. Rt, roots; ML, mature leaves; YL, young leaves; FC, female catkins; MC, male catkins; DX, differentiating xylems.

To gain a broader understanding of the function of LRR-RLKs, we analyzed the divergence among *Populus LRR-RLK* genes in spatial and temporal expression and expression in response to specific environmental signals. Probe sets were readily identifiable for 283 out of 379 PtLRR-RLKs in the PopGenExpress data set, and their distinct transcript abundance patterns were retrieved by the *Populus* Electronic Fluorescent Pictograph (eFP) browser [47]. Most *Populus* LRR-RLK genes demonstrated distinct tissue specific expression patterns except for mature leaves, where all have low transcriptional levels (Additional file 11). Filtering was added to select genes that had at least a 2-fold higher expression in one specific tissue compared to the median expression level of all analyzed tissues. Out of the *PtLRR-RLK* genes for which microarray data are available, 28%, 29%, 15%, 27% and 19% showed specific transcript accumulations in young leaf, roots, female catkins, male catkins and developing xylem, respectively (Figure 4A). Identification of the genes predominantly expressed in meristem tissues provides an important clue for their functions during cell fate specification and organ formation. Therefore, the expression of PtLRR-RLKs in multiple meristem tissues was investigated which may provide a further solid basis to select meristem-specific genes for related functional validation (Figure 4B).

Out of 16 tandem duplicated gene clusters, 8 clusters exhibited similar expression patterns among genes with expression data available (Additional file 12). It has been reported that in both rice and *Arabidopsis*, more than 50% of duplicate LRR-RLK gene pairs that were generated by a whole genome duplication event exhibited expressional divergence [48,49]. In poplar, among 82 pairs of *LRR-RLK* paralogs with expression data available, 68 (group I), 10 (group II) and 4 (group III) pairs shared >80%, 60-80% and <60% similarities over their full amino acid sequences, respectively. When expression patterns were compared, 70%, 30% and 0% pairs shared similar expression pattern in group I, II and III, respectively (Additional file 13). Thus, the expressional diversity of duplicated genes in poplar was correlated with the sequence variation which may represent a dynamic functional diversification of this gene family over evolutionary time and contribute to the adaptability of trees. Among the 63 *Arabidopsis* LRR-RLKs with known functions, 52 genes showed obvious tissue-specific expression instead of a whole-plant expression as illustrated by eFP and for 45 genes, their *Populus* homologues showed a similar spatial expression pattern (Additional file 14). This result supports that orthologous genes from different species may retain similar temporal and spatial expression patterns [50,51].

**Figure 5:**
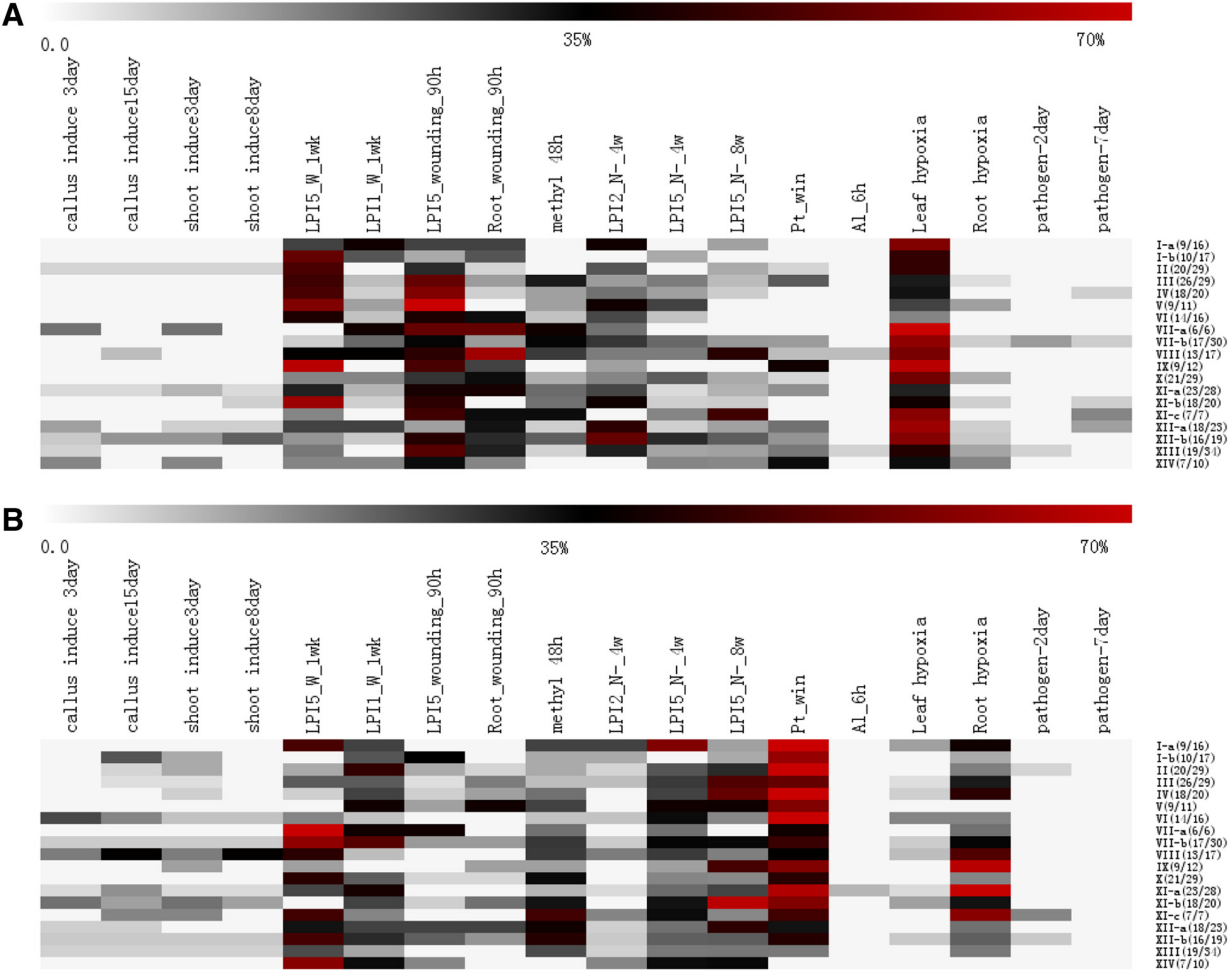
**Differential responsiveness of each *PtLRR-RLK *subfamily towards nine GEO (Gene Expression Omnibus) treatments.** The frequency of occurrence of *PtLRR-RLK* genes with up-regulation pattern (**A**) or down-regulation pattern (**B**) in each *PtLRR-RLK* subfamily is presented as a heat-map. Genome-wide transcriptome analysis include callus induction 3 day, callus induction 15 days, shoot induction 3 days and shoot induction 8 days (GSE12152, transcriptome analysis of shoot organogenesis in poplar); LPI5_W_1 wk and LPI1_W_1 wk (GSE16783, wound-induced gene expression changes in *Populus fremontii* × *Populus angustifolia*: 1 week; clone RM5); LPI5_W_90 h and Root_W_90 h (GSE16785, wound-induced gene expression changes in *Populus*: 90 hours); MJ_48 hr (GSE16773, gene expression response of *Populus tremuloides* cell suspension cultures to methyl jasmonate feeding); LPI2_N-_4w, LPI5_N-_4w, LPI5_N-_8w (*Populus* leaves under nitrogen limitation, GSE14893); Pt_winter (transcriptional regulation in the winter survival and maintenance mechanism of poplar, GSE21480); root tip, Al treated, at 6 h (Transcriptomic response to aluminum stress in roots of aspen (*Populus tremula* L., GSE19297); leaf hypoxia and root hypoxia (Effect of hypoxia on gene expression in Grey poplar, GSE13109); 3 day and 7 day (*Populus euphratica* leaves subjected to infection by *Marssonina* pathogen, GSE23726).

By complete searching of the digital expression profiles from the Gene Expression Omnibus (GEO) repository at NCBI website, we also investigated the expression patterns of the *PtLRR-RLK* genes during shoot organogenesis and in response to various stress stimulus including drought, cold, hypoxia, nitrogen limitation, aluminum stress in roots, bacteria, fungi and mimic wounding (Additional file 15). In a total of 12 treatments, the expression profile of *PtLRR-RLKs* varied considerably when exposed to 7 treatments, except for infection by *Marssonina* pathogen and *Melampsora* rust fungi, drought and aluminum stress in roots. Genes responsive to various treatments were summarized as heat maps in Additional file 16. The percentages of members of each subfamily being induced or suppressed for each treatment were listed in Additional file 17 and summarized in the format of heatmap in Figure 5. It can be seen that LRR-RLKs respond to various stimulates in a temporal and spatial manner by changing the expression profiles of different gene sets. For example, in wounding experiment, 90 hours after treatment (GSE16785), 102 and 59 *PtLRR-RLK* genes were up-regulated in leaf LPI5 and root, respectively, and qualitative differences in the induction patterns were detected for these two types of tissue (Figure 5). When the sampling time was extended to one week (GSE16783), only 31 and 87 genes were detected as induced in LPI1 leaves and LPI5 leaves, respectively, and compared to very young leaves (LPI1), the older leaves with LPI5 were much more enriched with up-regulated LRR-RLKs, which were overrepresented by members from subfamily III, IV, V, IX and XI-b (Figure 5). In another assay, the gene expression response of *Populus tremuloides* cell suspension cultures to methyl jasmonate feeding was analyzed; the transcript level of 37 *PtLRR-RLKs* was elevated. All these data indicated that LRR-RLK gene family plays an indispensable role in wounding defense of tree species. When confronted with ammonium shortage, at a 4-week checkpoint, the induction was more dramatic in young leaves (LPI2) than the older leaves (LPI5). With the progression of the ammonium shortage, the transcript of LRR-RLKs from subfamily III, IV, IX and XI-b got obviously repressed in the older leaves (Figure 5). When the effect of hypoxia on gene expression was investigated in grey poplar, 117 genes responded by induction in leaves with only 11 genes got induced in roots. This located induction pattern may imply the localized functions of different PtLRR-RLK members. From Figure 5, it was seen that members of different LRR-RLK subfamilies act in an overlapping manner when dealing with different stimulus which indicated that cross talk and signal integration exist among different signaling pathways mediated by PtLRR-RLKs. In terms of down-regulation, several things need to be pointed out (Figure 5B). First, members of VII and XIV subfamily were highly repressed in LPI5 leaves one week after wounding. Second, only 26 *PtLRR-RLK* genes got transcriptionally induced in the winter survival and maintenance mechanism of *P. trichocarpa*, 144 genes responded with repression instead (GSE21480). Third, in the hypoxia treatment, with more than 90% of the induced genes was located in the leaf tissue, 88% down-regulated genes were found in the root tissue instead. In summary, although it is hard to assign distinct roles to different *Populus* LRR-RLK subfamilies based on the results of limited microarray analysis, it could be reasonable to suggest that PtLRR-RLKs are widely involved in different aspects of plant development in both normal and stressed circumstances. However, subset of *Arabidopsis LRR-RLK* genes have previously been shown to play crucial roles in biotic stress response (Additional file 18). Two biotic signals, *Marssonina* pathogen and *Melampsora* rust fungi, did not cause significant change of gene expression profiles in the current study, which indicates a need for more microarray experiments to better understand the roles of *Populus LRR-RLK* genes in biotic defense. For trees, it is unlikely to generate a collection of LRR-RLK T-DNA insertion mutants, as in *Arabidopsis,* to be easily applied for the analysis of other developmental aspects. The results from this study could provide insights into possible functions for some PtLRR-RLKs before future functional analyses would eventually elucidate their biological meanings.

## Conclusions

Characterization of LRR-RLK genes in a ligneous species would facilitate a better understanding of the evolutionary processes and functions of this gene family. The current work shows that the LRR-RLKs represent a large gene family in *Populus trichocarpa*. Gene structures, motif composition and arrangements are considerably conserved among the (sub)groups. The distribution of genes was found to be non-random across chromosomes and a high proportion of the genes are located in segmental duplicated regions instead of tandem duplicated clusters. For most of the 63 *Arabidopsis* with known functions, *Populus* homologues always could be identified with similar genetic structure, motif character and expression profiles, providing insight into the evolutionary and functional conservation of this gene family in plant species. Expression patterns based on microarray data suggest that many PtLRR-RLK genes are expressed in a tissue-specific manner and responsive to various stresses. Data in this work may provide valuable information for future investigations to reveal the functional divergence and adaptive evolution of this gene family in tree species.

## Methods

### Sequence retrieval and phylogenetic reconstruction of LRR-RLK genes in poplar genome

*Arabidopsis thaliana* gene identifiers of different LRR-RLK super-families were downloaded from the PlantsP server v.2011 *Arabidopsis* 2010 project (http://plantsp.genomics.purdue.edu/html/projects/lrr/Clouse2010.htm) [5] for the first round Blastp search against the poplar genomic sequence database at the DOE Joint Genome Institute (JGI) website. Subsequently, each identified hit was used as a query to conduct Blastp searches in the poplar assembly genomic sequence database to minimize the risk of missing potential PtLRR-RLKs. The version 2.2 *P. trichocarpa* genome and protein sequences were downloaded from Phytozome (http://www.phytozome.net) [52]. These resulted hit sequences were then analyzed with SMART (http://smart.embl-heidelberg.de) [53] and PFAM (http://pfam.sanger.ac.uk/) [54] to assure the presence of at least two LRR domains and one RLK domain. Identical and defective sequences were eliminated using manual inspection in Molecular Evolutionary Genetics Analysis (MEGA) v5.1 [55]. After the signal sequences were deleted, ClustalX v.2.0.12 [56] was used to generate a multiple sequence alignment of either the full length sequences, the trimmed LRR domains or kinase domains among the PtLRR-RLK protein sequences. The phylogenetic trees were constructed using the neighbor-joining method [57] in the MEGA package v5.1 [55] with bootstrap values from 1000 replicates indicated at each node. Representative sequences from each *Arabidopsis* LRR-RLK subfamily or AtLRR-RLKs with defined functions were chosen to generate alignments with *Populus* LRR-RLKs.

### Protein structure and conserved motif distribution

The number and position of exons and introns for individual *PtLRR-RLK* genes were determined by comparison of the cDNAs with their corresponding genomic DNA sequences. Information concerning PtLRR-RLK protein sequences, such as number of amino acids, molecular weights and PIs, were determined using ProtParam (http://au.expasy.org/tools/protparam.html) [58]. Presence of the signal peptides were predicted at SignalP v.4.1 (http://www.cbs.dtu.dk/services/SignalP) [59]. Transmembrane domains were predicted with TMHMM v. 2.0 (http://www.cbs.dtu.dk/services/TMHMM-2.0/) [60] and Phobius (http://phobius.binf.ku.dk/) [61]. To exhibit the structural divergence of PtLRR-RLK genes, the conserved motifs in the encoded proteins were performed with the Multiple Expectation Maximization for Motif Elicitation (MEME) online program v.4.9.0 (http://meme.sdsc.edu/meme/intro.html) [37] and visualized with WebLogo (http://weblogo.berkeley.edu/logo.cgi) [62]. Parameters were set as follows: the maximum number of motifs 30; minimum motif width 10; and maximum motif width 30; all other parameters were defaulted.

### Chromosome location analysis

The chromosomal locations of the poplar LRR-RLK genes were drawn on the schematic diagram tool at PopGenIE [63] (http://popgenie.org/gp). Identification of homologous chromosome segments resulting from whole-genome duplication events was accomplished as described previously [17]. Blocks with the same color represent homologous chromosome segments. Tandem gene duplications were identified as genes separated by ten or fewer gene loci in a range of 200 kb distance.

### Gene expression analysis

Gene expression data mainly came from Poplar eFP Browser (http://bar.utoronto.ca/efppop/cgi-bin/efpWeb.cgi). In addition, the gene expression pattern of *Populus* meristem tissue series was obtained from PopGenIE [63]. The genome-wide microarray data was obtained from the Gene Expression Omnibus database at the NCBI under the series accession numbers GSE23637 (*Populus euphratica* leaves subjected to drought), GSE13043 (from *P. trichocarpa*), GSE21480 (transcriptional regulation in the winter survival), GSE20061 (young differentiating xylem of poplar in response to a drought –rewatering cycle), GSE23726 (*Populus euphratica* leaves subjected to infection by Marssonina pathogen), GSE9673 (interactions with *Melampsora* rust fungi), GSE13109 (Effect of hypoxia on gene expression in Grey poplar), GSE14893 (*Populus* leaves under nitrogen limitation: clone 3200), GSE19297 (aluminum stress in roots of aspen, *Populus tremula* L.), GSE16773 (gene expression response of *Populus tremuloides* cell suspension cultures to methyl jasmonate feeding), GSE12152 (Genome scale transcriptome analysis of shoot organogenesis in *Populus tremula* x *P. alba*), GSE17223 (Molecular bases of acclimation and adaptation to water deficit in *Populus anadensis*) and GSE16785 (Wound-induced gene expression changes in *Populus*: 90 hours; clone RM5). Probe sets corresponding to the putative *Populus* LRR-RLKs were identified using an online Probe Match tool available at the NetAffx Analysis Center (http://www.affymetrix.com/). Genes were clustered based on the expression profiles and Hierarchical clustering of microarray data performed in MultiExperiment Viewer (MeV) v4.7.4 [64], using Pearson correlation and Average Linkage Clustering algorithm. Heatmaps of gene expression were generated using R (http://www.r-project.org/).

## Competing interests

The authors declare that they have no competing interests.

## Authors’ contributions

YZ and YJ performed most of the data mining and data analysis. YZ participated in the illustrations of the figures and tables. SY and YS helped to retrieve data from GEO database and draw the heat-map. JW designed and coordinated the work and wrote the manuscript. All authors read and approved the final manuscript.

## Supplementary Material

Additional file 1 **A complete list of 379 *PtLRR-RLKs* identified in the present study.** Genomic DNA sequences are obtained from Phytozome (http://www.phytozome.net/poplar, release 2.1). Amino acid sequences are deduced from the corresponding coding sequences. Click here for file

Additional file 2 **Maximum likelihood bootstrap tree phylogeny based on the LRR sequences of *LRR-RLK* genes in *Populus trichocarpa.*** The unrooted tree was constructed using MEGA 4.0. Numbers at nodes indicate the percentage bootstrap scores and only bootstrap values higher than 50% from 1,000 replicates are shown. Click here for file

Additional file 3 **Maximum likelihood bootstrap tree phylogeny based on the RLK sequences of *LRR-RLK* genes in *Populus trichocarpa.*** The unrooted tree was constructed using MEGA 4.0. Numbers at nodes indicate the percentage bootstrap scores and only bootstrap values higher than 50% from 1,000 replicates are shown. Click here for file

Additional file 4 **Maximum likelihood bootstrap tree phylogeny based on the LRR sequences of *LRR-RLK* genes in *Populus trichocarpa *and *Arabidopsis thaliana.*** The unrooted tree was constructed using MEGA 4.0. Numbers at nodes indicate the percentage bootstrap scores and only bootstrap values higher than 50% from 1,000 replicates are shown. Click here for file

Additional file 5 **The JGI gene model of each *PtLRR-RLK* gene to illustrate the distribution and position of introns.** Exons and introns are represented to scale by colored boxes and lines, respectively. The group number and name of *PtLRR-RLK* gene and its intron–exon structure pattern are indicated at the left and right sides, respectively. Click here for file

Additional file 6 **Comparison of intron/exon structures between *AtLRR-RLKs* with known functions and *Populus* homologues with similar genetic structures.** Click here for file

Additional file 7 **Schematic illustrations of the types and distributions of LRR motifs in each *Populus* LRR-RLK subfamily.** Click here for file

Additional file 8 Non-LRR motifs identified in the extracellular regions of PtLRR-RLKs. Click here for file

Additional file 9 **PtLRR-RLKs sharing the same or similar motif composition and arrangement as AtLRR-RLKs (50 out of 63 with known functions).** The motif characterization was based on full-length proteins. Click here for file

Additional file 10 Locations of tandem clustering on chromosomes and their distribution among fourteen phytogenetic groups. Click here for file

Additional file 11 **Tissue-specific expression patterns of 283 out of 379 *PtLRR-RLKs* with probes available in the PopGenExpress data set.** The microarray-based expression data were downloaded from the Poplar eFP browser, gene-wise normalized and hierarchical clustered based on Pearson correlation. Color scale at the top of each dendrogram represents log2 expression values. Rt, roots; ML, mature leaves; YL, young leaves; FC, female catkins; MC, male catkins; DX, differentiating xylems. Click here for file

Additional file 12 **Expression patterns of tandem duplicated gene clusters.** The microarray-based expression data were downloaded from the Poplar eFP browser, gene-wise normalized and hierarchical clustered based on Pearson correlation. Color scale at the top of each dendrogram represents log2 expression values. Rt, roots; ML, mature leaves; YL, young leaves; FC, female catkins; MC, male catkins; DX, differentiating xylems. Click here for file

Additional file 13 **Expression patterns of 82 pairs of PtLRR-RLK paralogs.** The microarray-based expression data were downloaded from the Poplar eFP browser, gene-wise normalized and hierarchical clustered based on Pearson correlation. Color scale at the top of each dendrogram represents log2 expression values. Rt, roots; ML, mature leaves; YL, young leaves; FC, female catkins; MC, male catkins; DX, differentiating xylems. Click here for file

Additional file 14 **Comparison of tissue expression patterns of *Arabidopsis* LRR-RLKs and their respective *Populus* homologues.** Click here for file

Additional file 15 Description of Gene Expression Omnibus (GEO) datasets at NCBI website used in this study. Click here for file

Additional file 16 ***Populus* LRR-RLK genes exhibit differential expression upon a range of treatments.** The patterns of relative transcript accumulation of each PtLRR-RLK genes as determined by microarray analysis are presented as a heat map, with red indicating higher levels and blue indicating lower levels of transcript accumulation. Click here for file

Additional file 17 The percentages of PtLRR-RLK genes from each subfamily being induced (A) or suppressed (B) for each treatment. Click here for file

Additional file 18 Information on the AGI code, gene full name and abbreviation for each AtLRR-RLK gene with defined functions presented in this work. Click here for file
