# Supplementary figures and images for: Genome-wide identification, characterization and expression analysis of populus leucine-rich repeat receptor-like protein kinase genes
Source: BMC Genomics. 2013 May 10;14:318. doi: 10.1186/1471-2164-14-318 (PMC3682895; doi:10.1186/1471-2164-14-318)

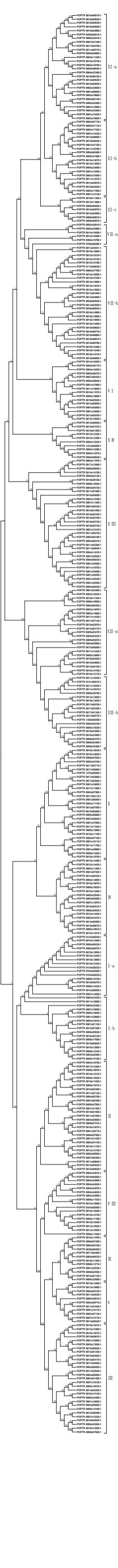

Supplement: Additional file 2 — Maximum likelihood bootstrap tree phylogeny based on the LRR sequences of LRR-RLK genes in Populus trichocarpa. The unrooted tree was constructed using MEGA 4.0. Numbers at nodes indicate the percentage bootstrap scores and only bootstrap values higher than 50% from 1,000 replicates are shown. [file 1471-2164-14-318-S2.pdf]

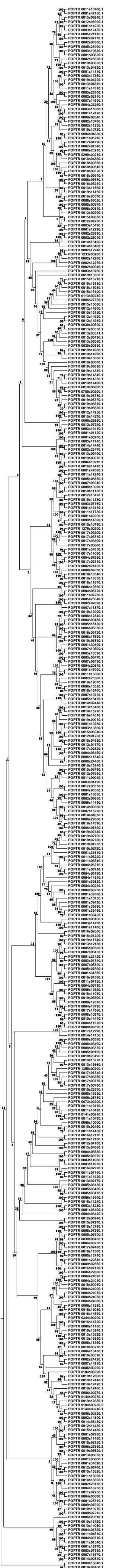

Supplement: Additional file 3 — Maximum likelihood bootstrap tree phylogeny based on the RLK sequences of LRR-RLK genes in Populus trichocarpa. The unrooted tree was constructed using MEGA 4.0. Numbers at nodes indicate the percentage bootstrap scores and only bootstrap values higher than 50% from 1,000 replicates are shown. [file 1471-2164-14-318-S3.pdf]

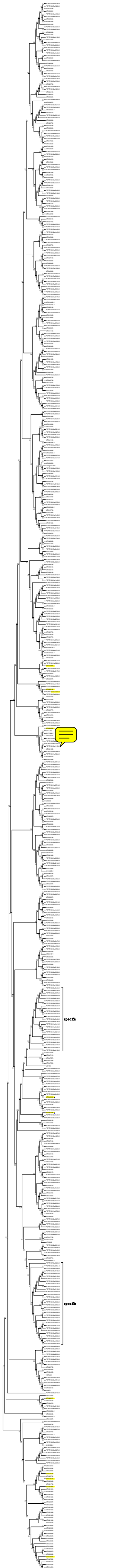

Supplement: Additional file 4 — Maximum likelihood bootstrap tree phylogeny based on the LRR sequences of LRR-RLK genes in Populus trichocarpa and Arabidopsis thaliana. The unrooted tree was constructed using MEGA 4.0. Numbers at nodes indicate the percentage bootstrap scores and only bootstrap values higher than 50% from 1,000 replicates are shown. [file 1471-2164-14-318-S4.pdf]

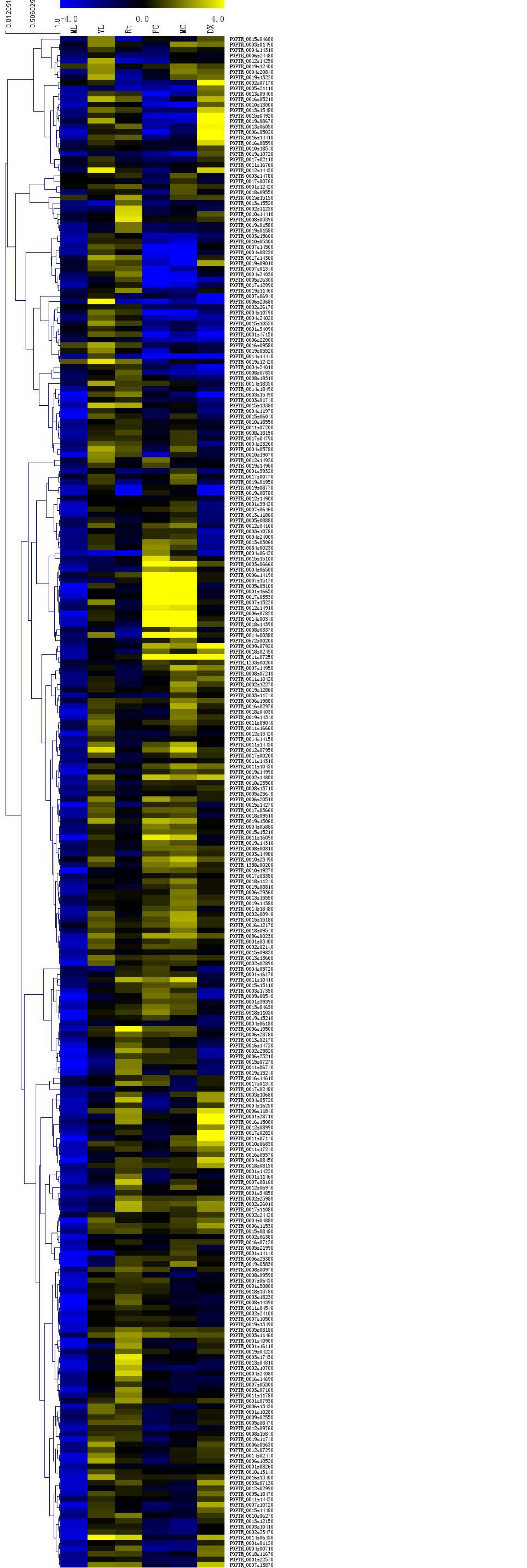

Supplement: Additional file 12 — Expression patterns of tandem duplicated gene clusters. The microarray-based expression data were downloaded from the Poplar eFP browser, gene-wise normalized and hierarchical clustered based on Pearson correlation. Color scale at the top of each dendrogram represents log2 expression values. Rt, roots; ML, mature leaves; YL, young leaves; FC, female catkins; MC, male catkins; DX, differentiating xylems. [file 1471-2164-14-318-S12.pdf]

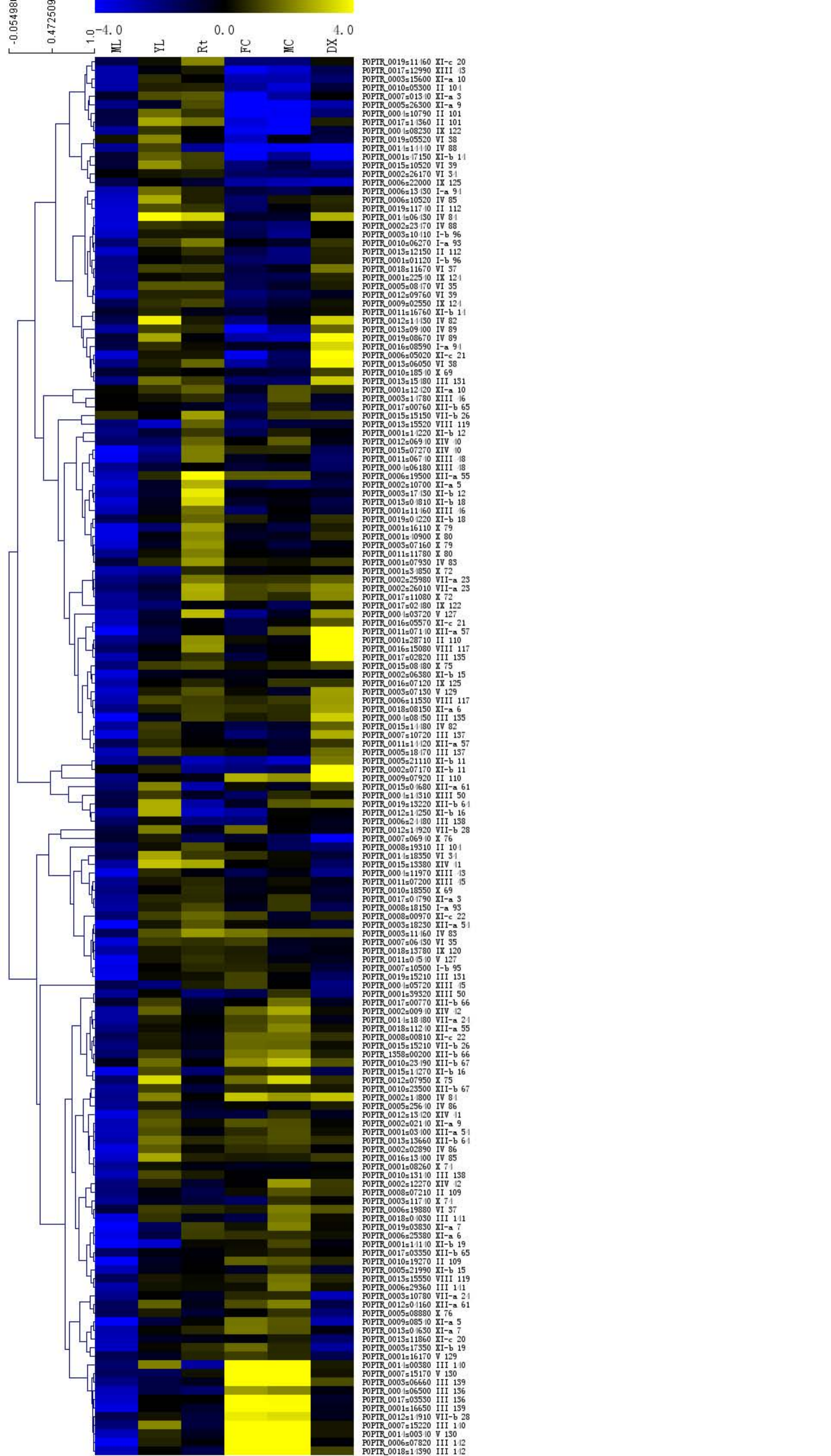

Supplement: Additional file 13 — Expression patterns of 82 pairs of PtLRR-RLK paralogs. The microarray-based expression data were downloaded from the Poplar eFP browser, gene-wise normalized and hierarchical clustered based on Pearson correlation. Color scale at the top of each dendrogram represents log2 expression values. Rt, roots; ML, mature leaves; YL, young leaves; FC, female catkins; MC, male catkins; DX, differentiating xylems. [file 1471-2164-14-318-S13.pdf]

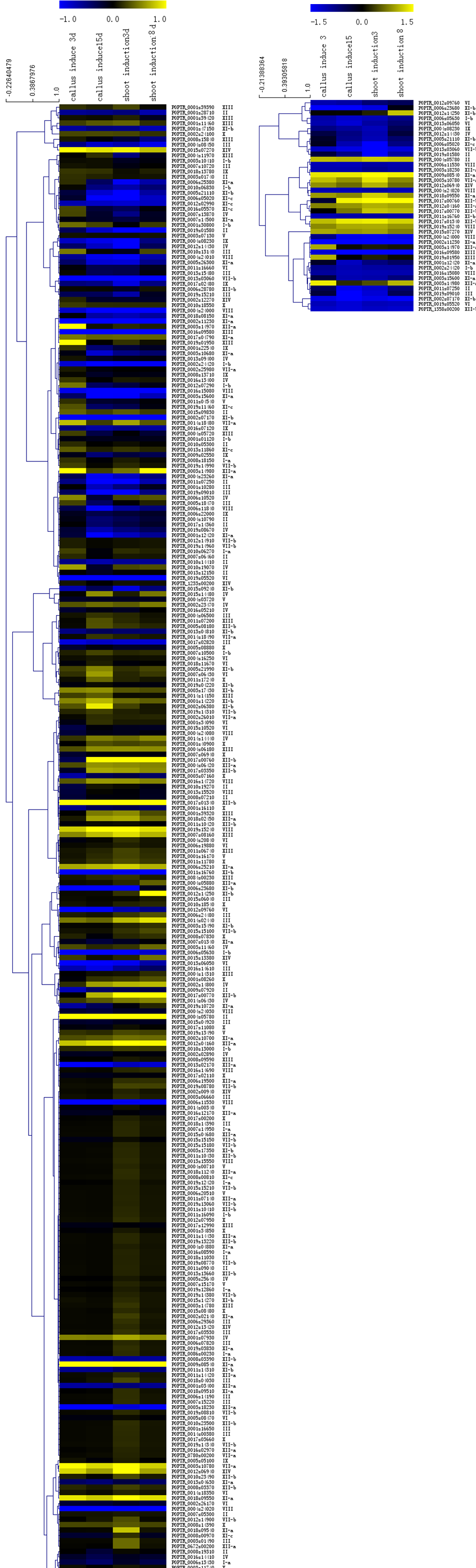


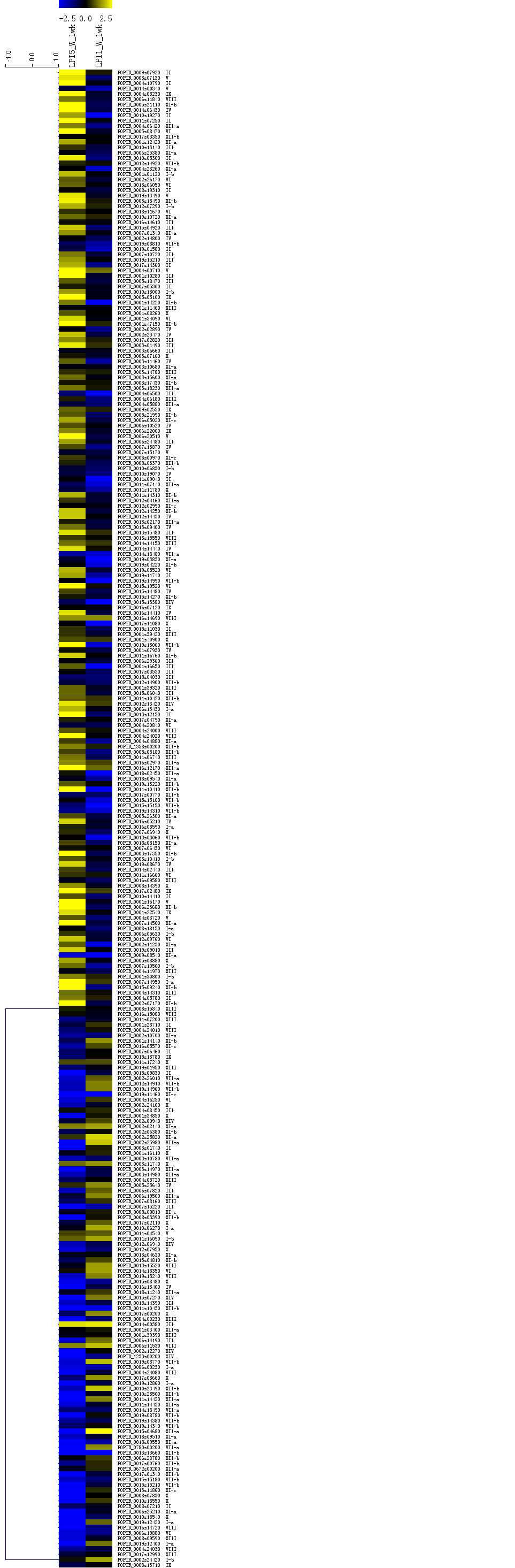


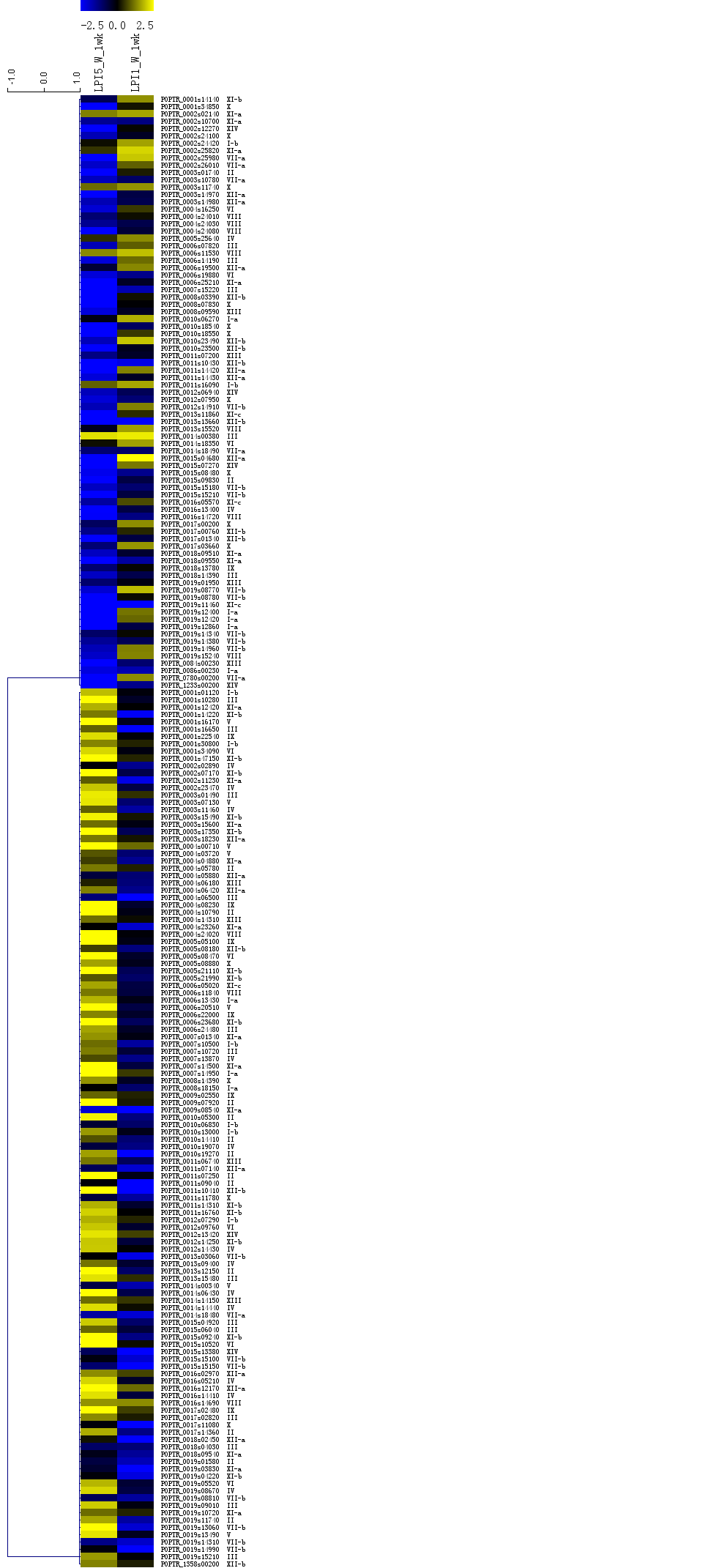


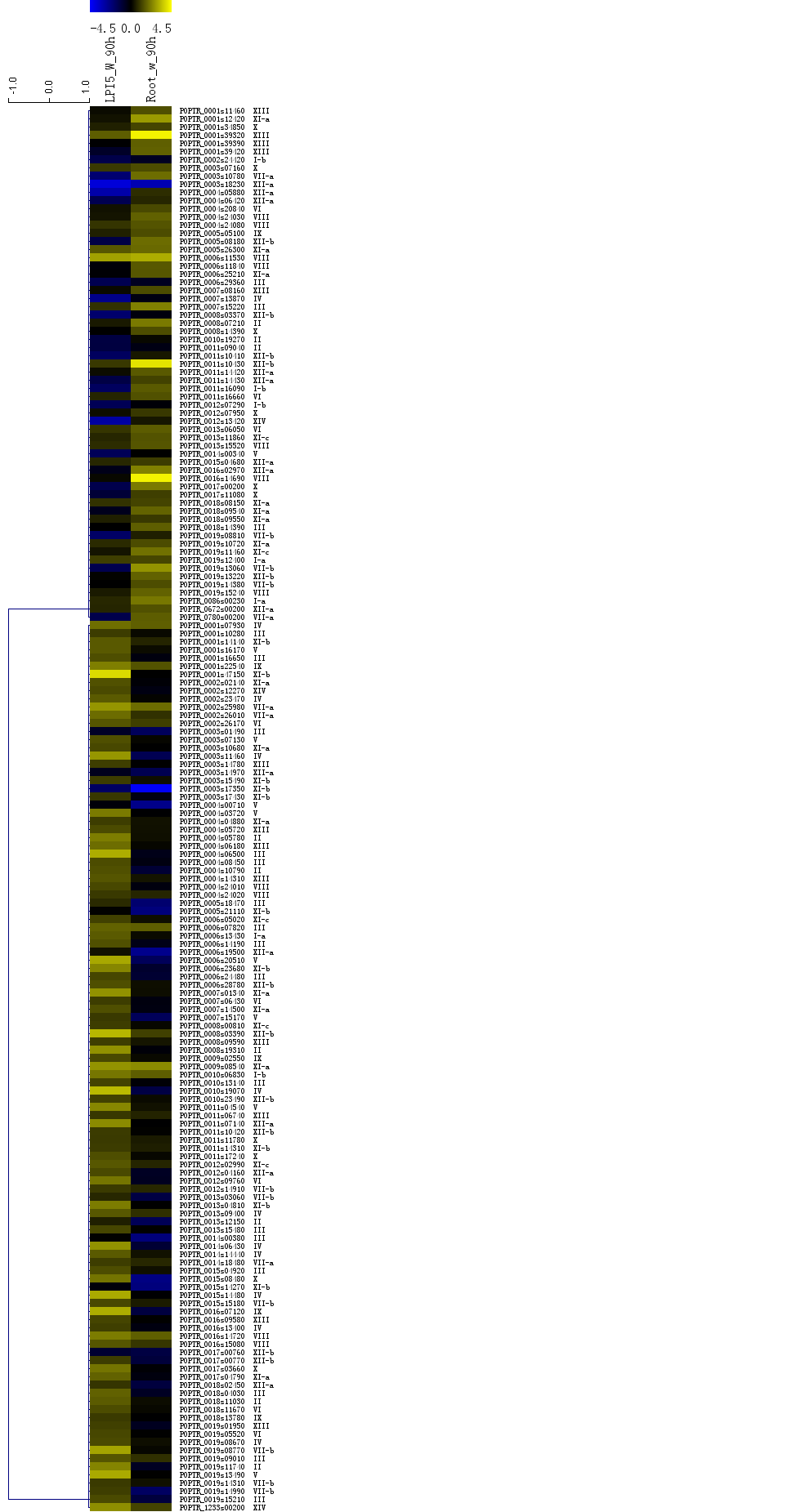


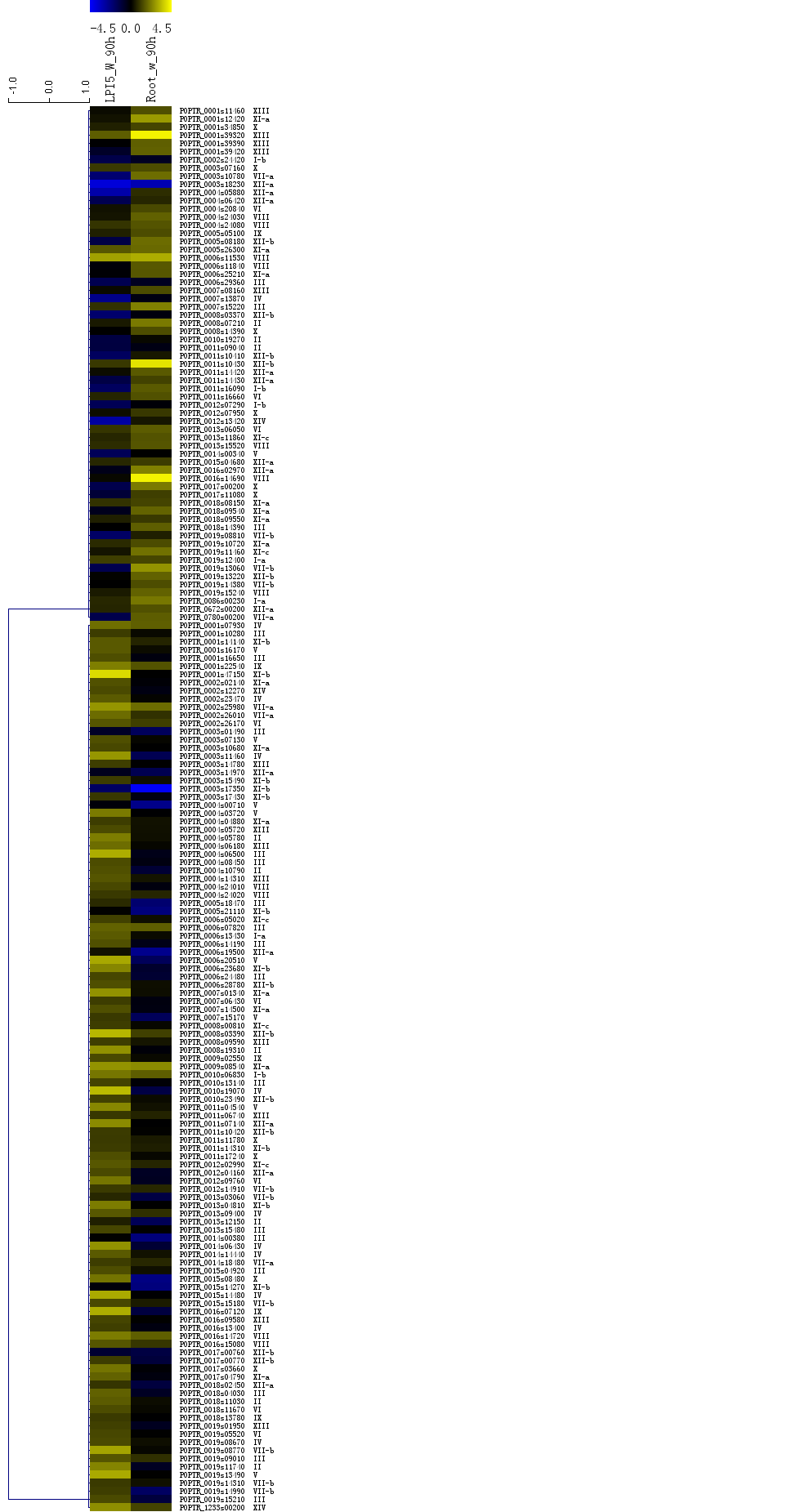


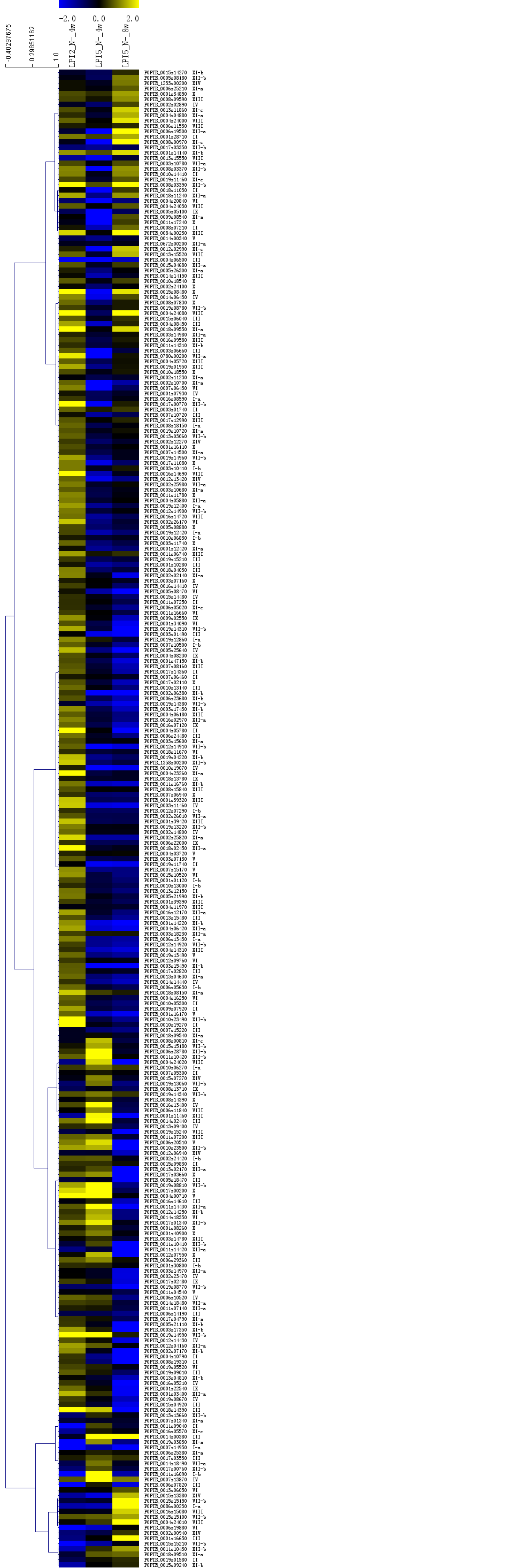


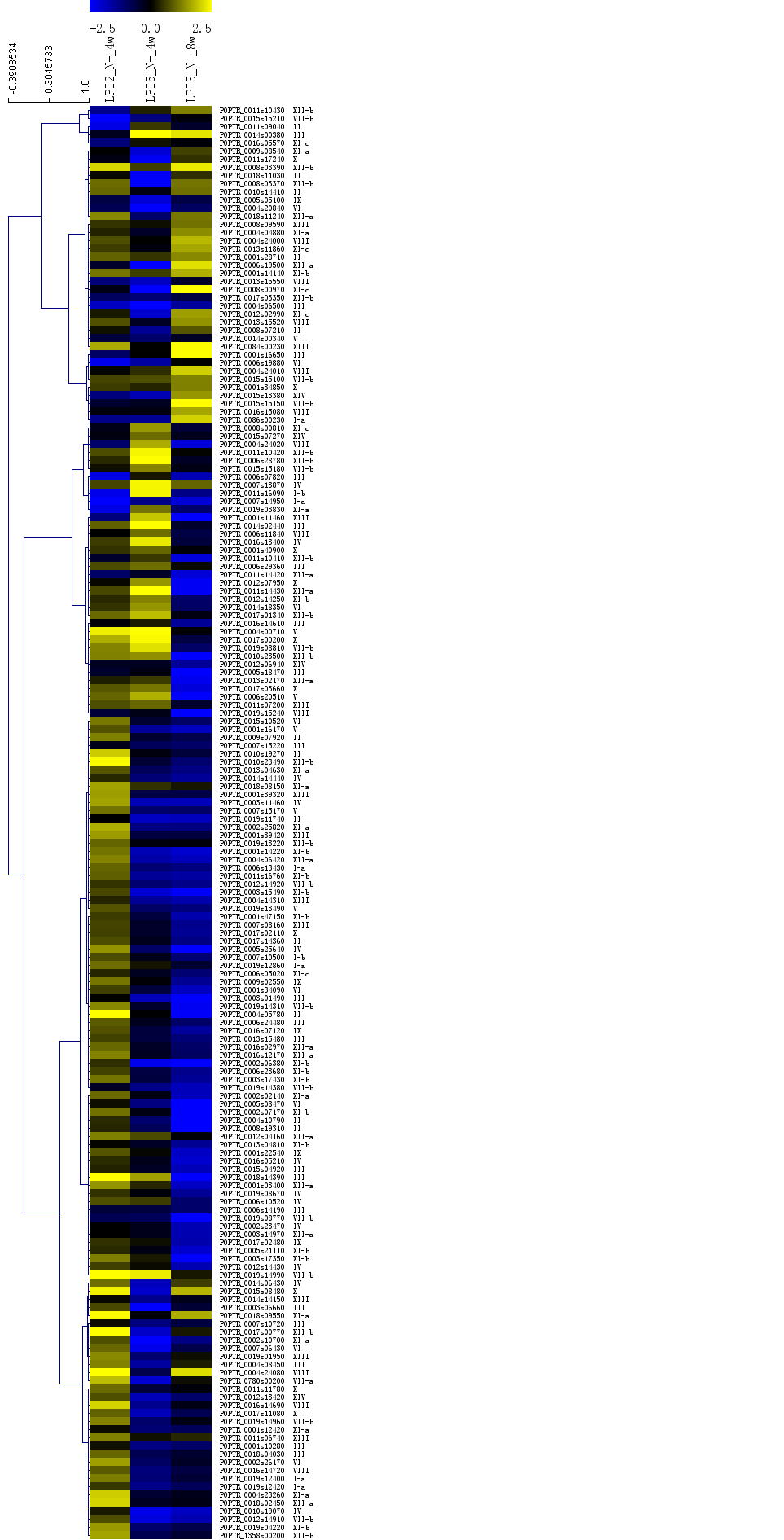


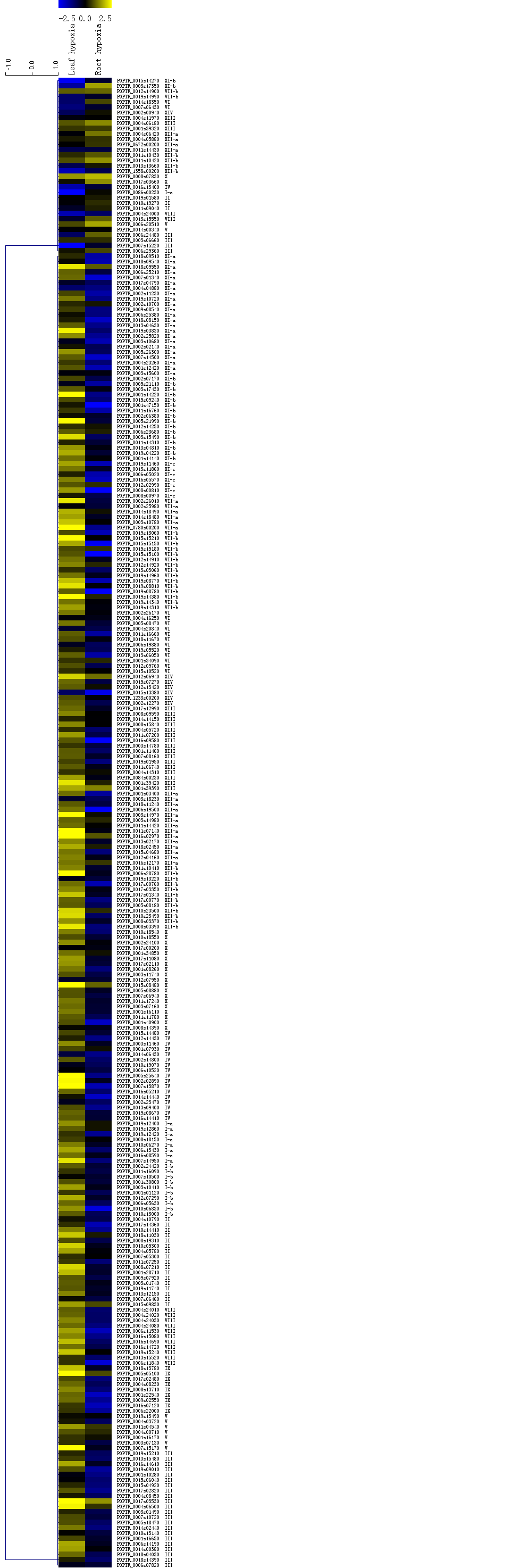


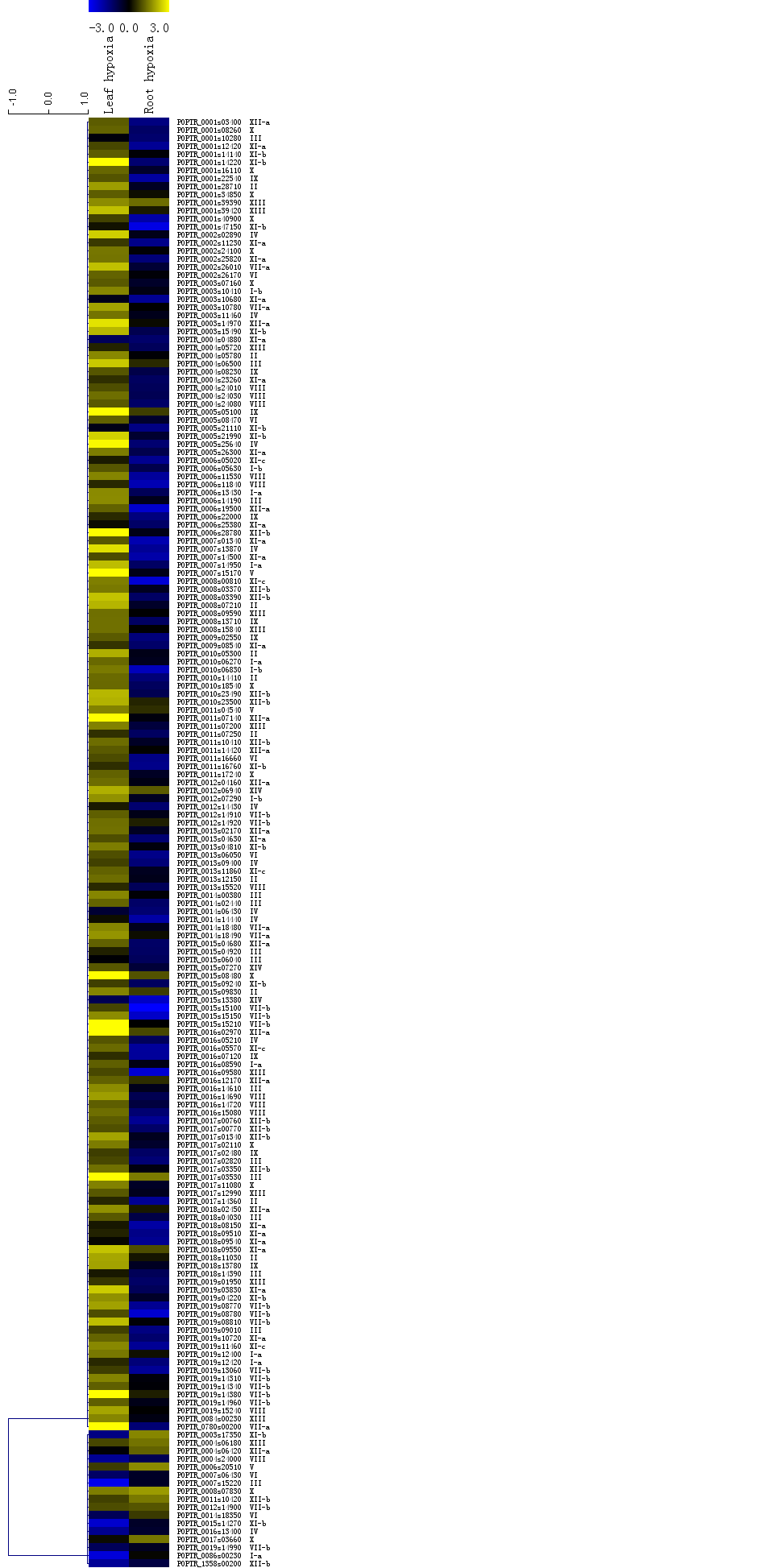


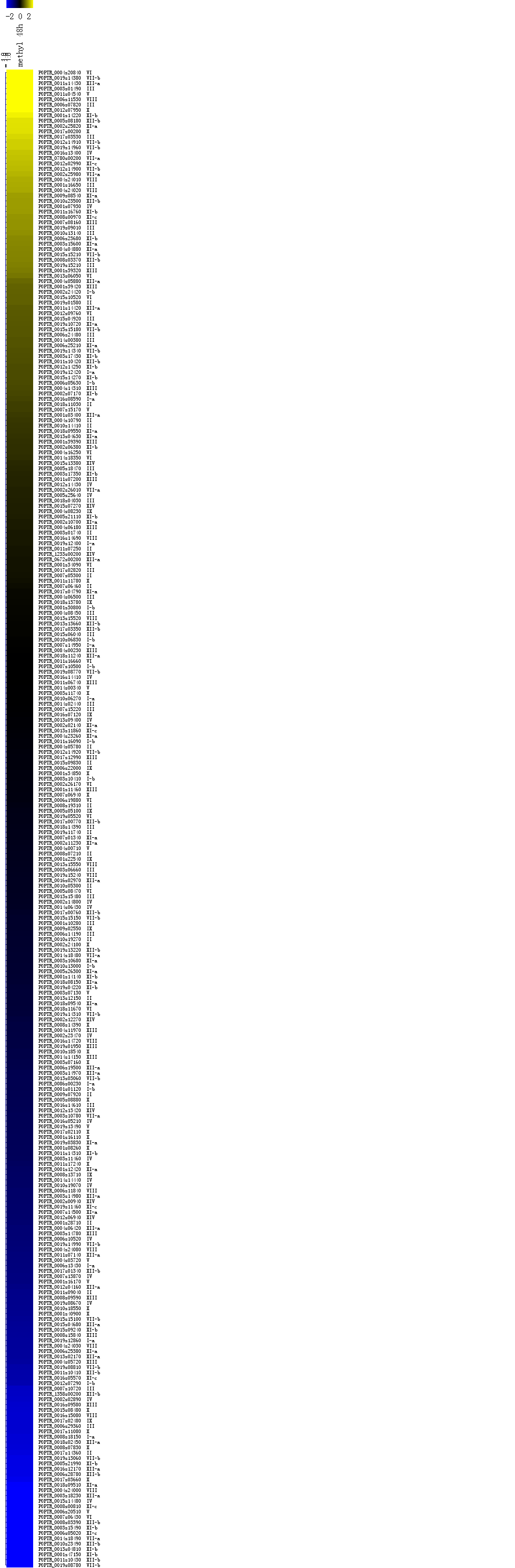


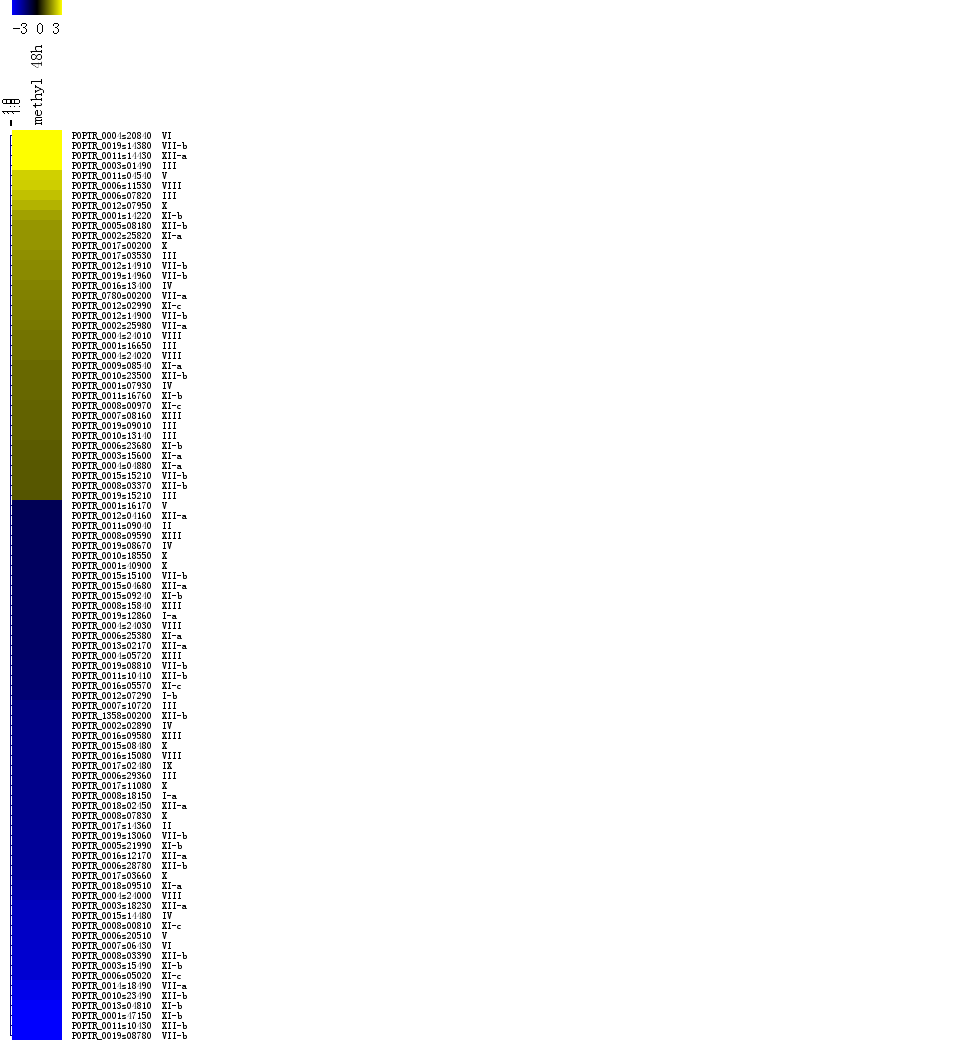


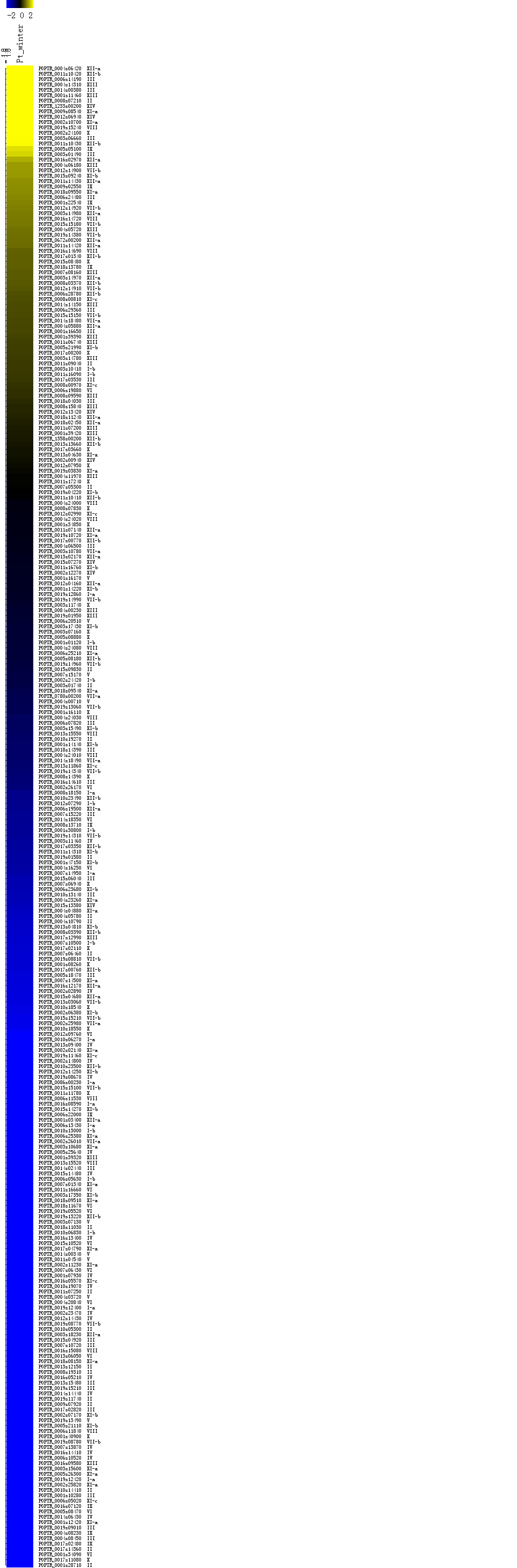


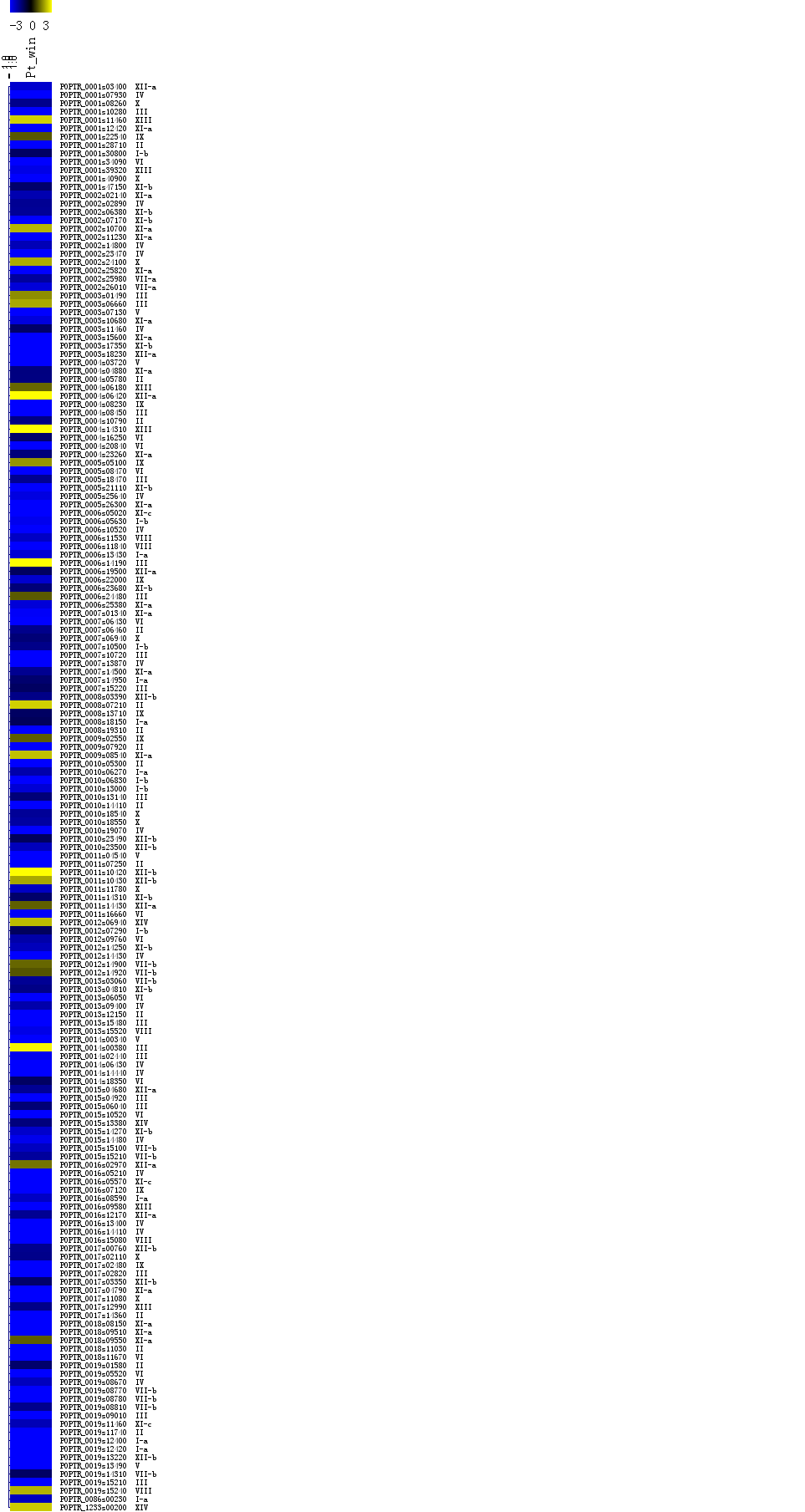


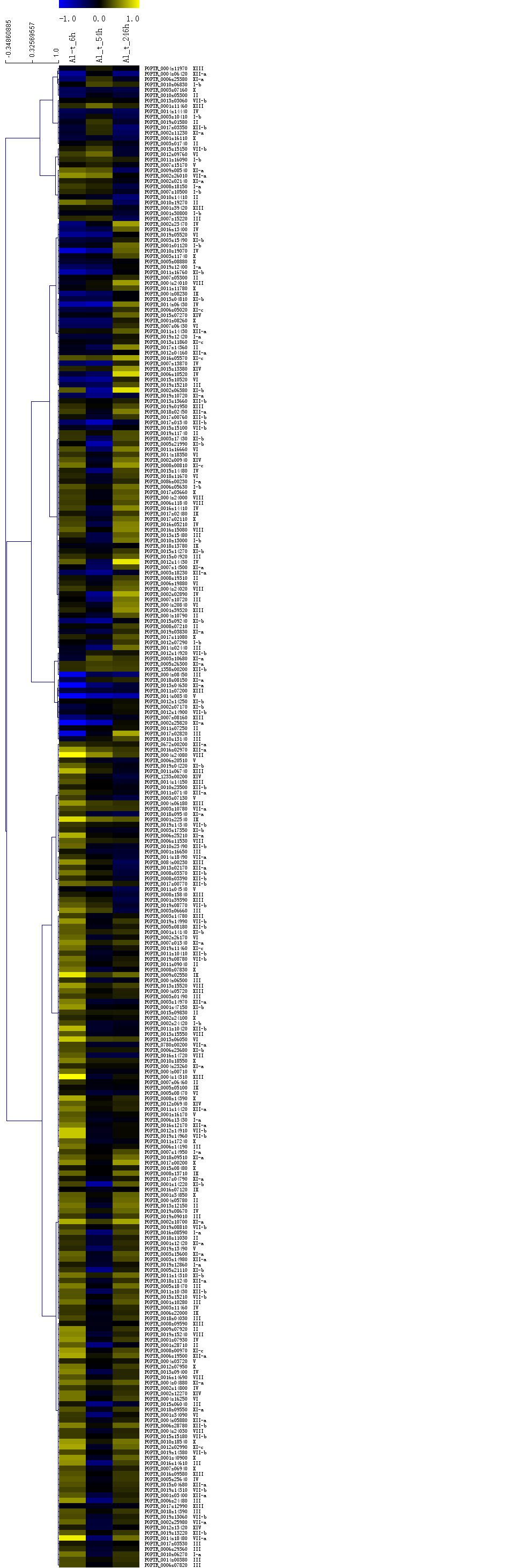


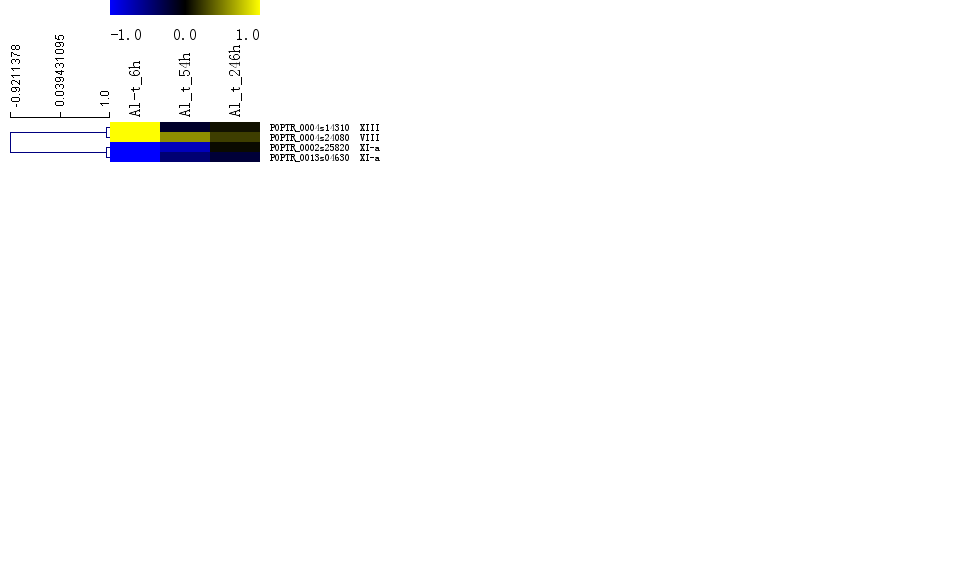


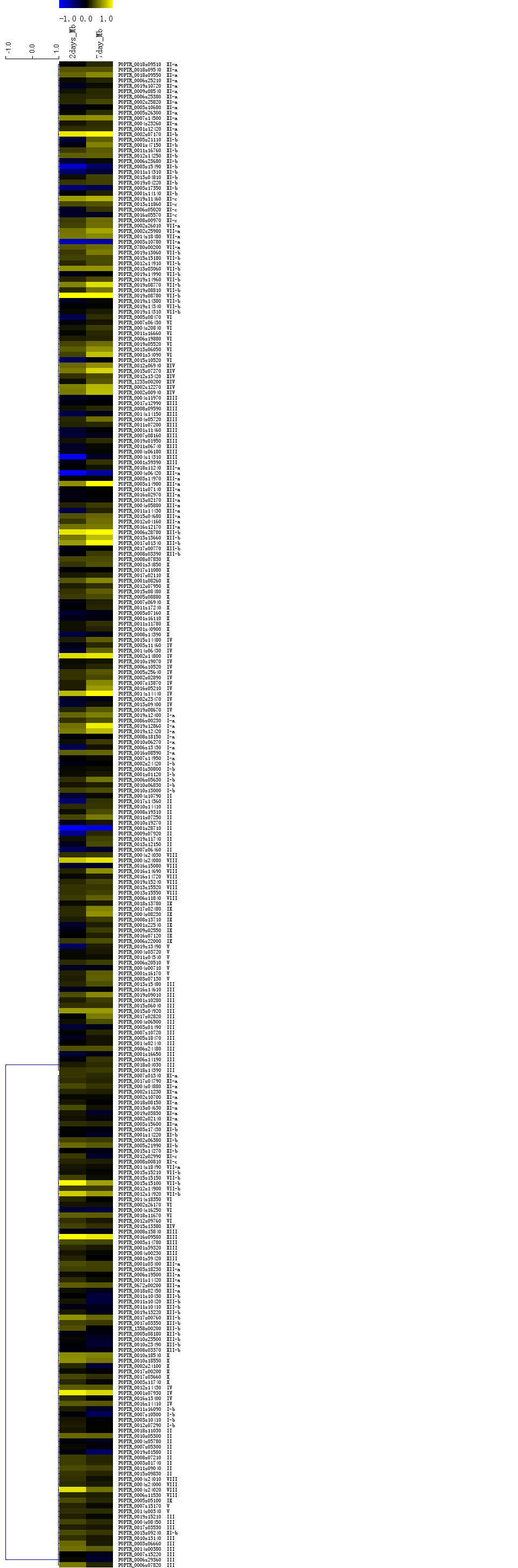


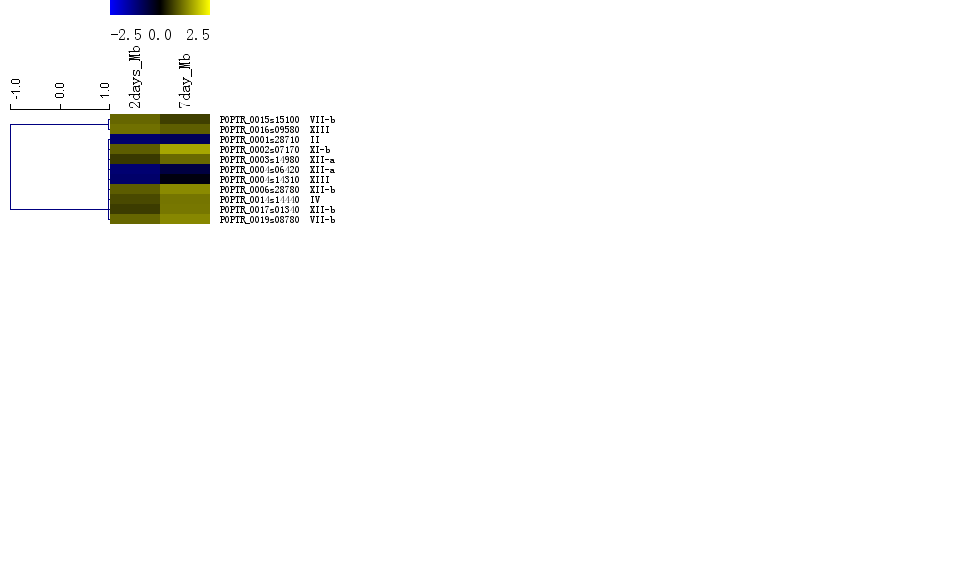


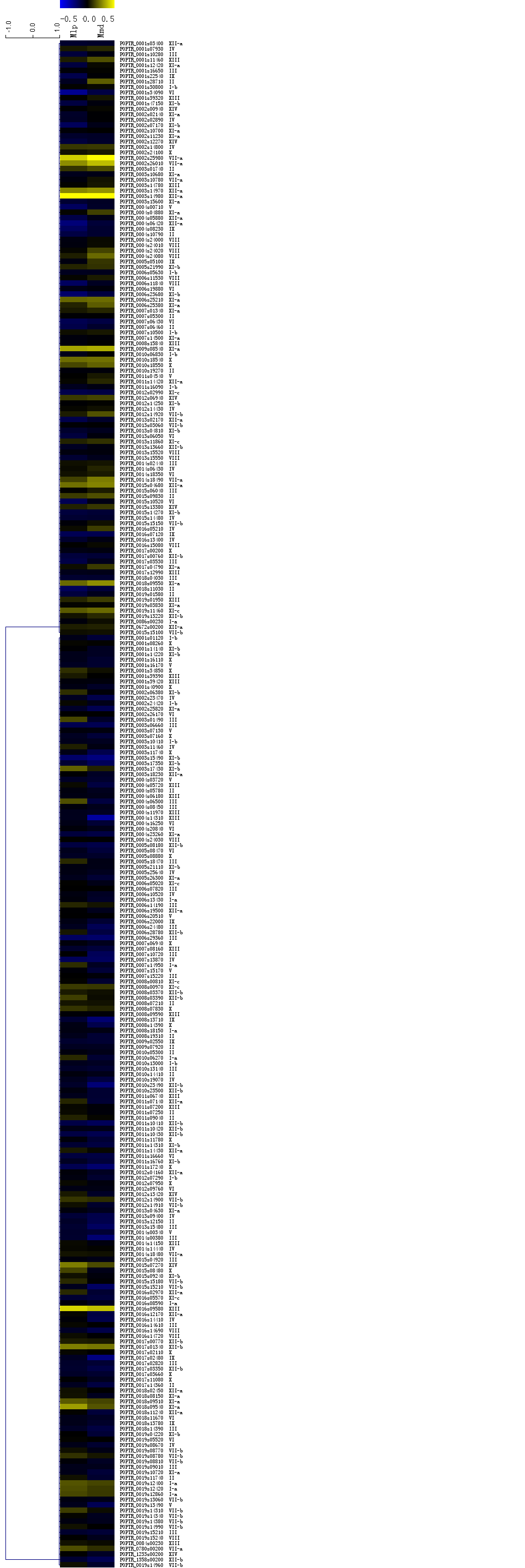


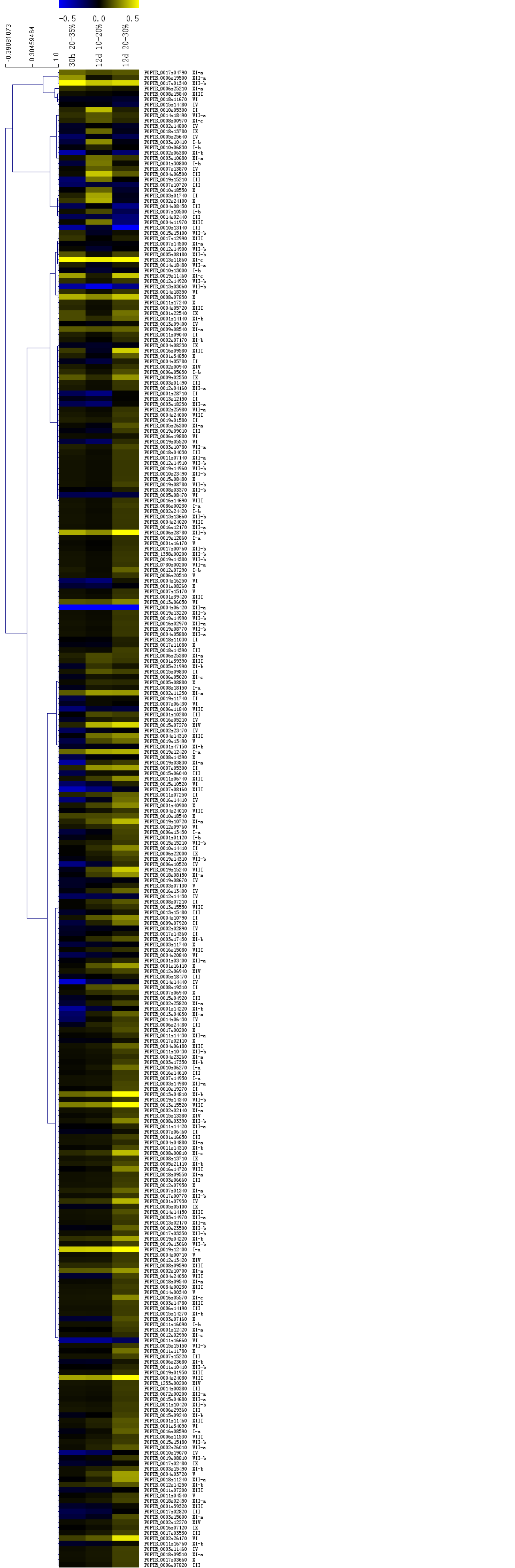


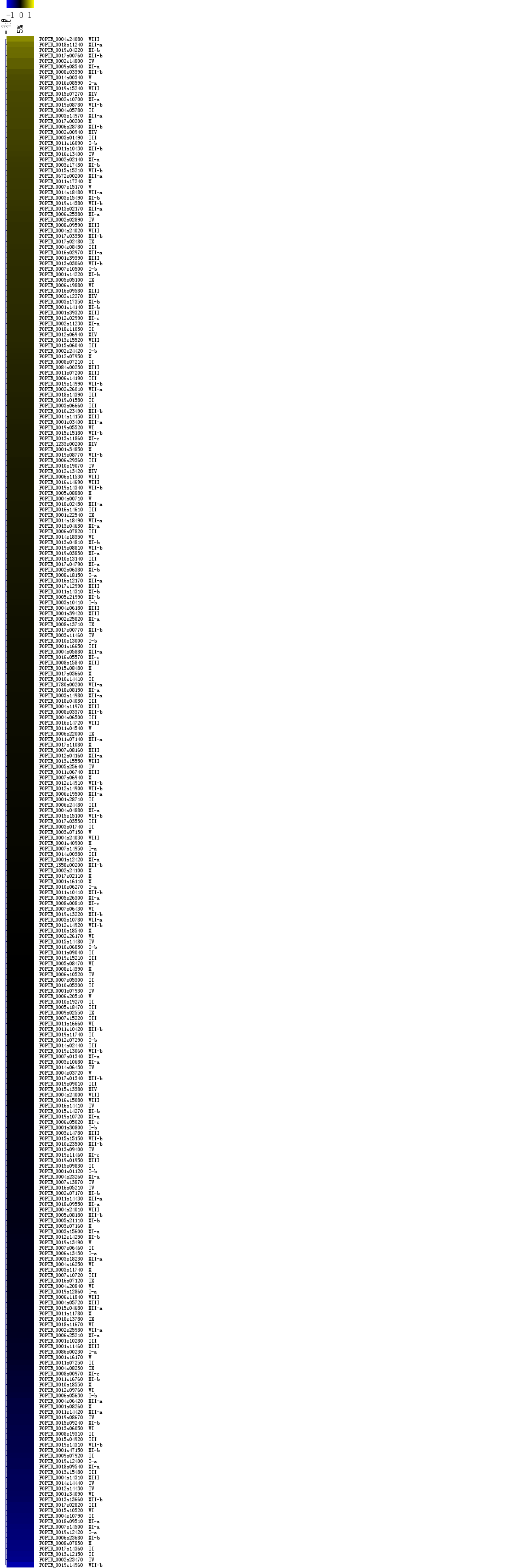

Supplement: Additional file 16 — Populus LRR-RLK genes exhibit differential expression upon a range of treatments. The patterns of relative transcript accumulation of each PtLRR-RLK genes as determined by microarray analysis are presented as a heat map, with red indicating higher levels and blue indicating lower levels of transcript accumulation. [file 1471-2164-14-318-S16.doc]
