# Supplementary material for: Genome-wide identification, characterization and expression analysis of populus leucine-rich repeat receptor-like protein kinase genes
Source: BMC Genomics. 2013 May 10;14:318. doi: 10.1186/1471-2164-14-318 (PMC3682895; doi:10.1186/1471-2164-14-318)
Supplement: Additional file 8 — Non-LRR motifs identified in the extracellular regions of PtLRR-RLKs. [file 1471-2164-14-318-S8.doc]

| Pt M4 | xxx**x**xxxxp**C**x**W**x**G** V/I x**C** |
| --- | --- |
| Pt M13 | **s**SF**x**G**N**P**gLCG**x**P**L |
| Pt M14 | xnx D/E xxA**L**Ls F/l **K**xx L/I xxpx |
| Pt M17 | **V/l** xxL**L**xxxx**L**xG |
| Pt M21 | **AR L**/v **A**/s **P** Q/a**SL K**/t **YY**g **L**/r **C** M/L L/p/e A/n/k **G** s/n/k **Y K**/t **V** Q/s **LHFAE** |
| Pt M22 | **E**/d **I H**/r **LYW** S/A **GKGTT**a V/I **P**D **R**/s **G** V/n **TGP L**/I **ISAI** |
| Pt M24 | **S** S/P P N/p **C** K/f **CA**x**P** Y/l T G/I xLx **F**/I **R** A/s **PSFS** |
| Pt M25 | **V**/c C/i/l xx K/l P/a Q/D **Y**/w **H**/r **S** L/m/f F/y/h **INCGG** S/Q S/n V/E T/K V/t/n **G**/n DNV**YE** |
| Pt M26 | **I**/t **KAF**R E/N **A P**/S **F**/y **EA**I**R** N/D **N** |
| Pt M27 | **C D**/h A/p Q/t/r **V**x **T**/v **LL**S **I**/v **A**/v K/G G/n **F**/m **G**/y **YP**xx**L** S/A **D**g**W K**/d **G**/d **N** D/n |
| Pt M28 | **C** K/r/h **G** V/e/a **G G**/n **L L**/v **EF A**/q **GI** R/K **A**/w **E R**/q **L** L/n/e Q/r/n V/l/i **P**/s T/s/m s/l/f/a K/H T/s/p **C** D/s/n **F**/t **T**/s |
| Pt M29 | **Y Y**/q **T**/R V/p/l **L**/V **E**/q q/k/e/a **S**/a **L**/M **M** r/n/e/d **SFK** s/n/l/f H/s/d **Q**/S **LPVDS V**/I N/y/s **L**/v **S**/n H/v/c **P** R/t/l **K**/N |
