# Supplementary material for: Genome-wide identification, characterization and expression analysis of populus leucine-rich repeat receptor-like protein kinase genes
Source: BMC Genomics. 2013 May 10;14:318. doi: 10.1186/1471-2164-14-318 (PMC3682895; doi:10.1186/1471-2164-14-318)
Supplement: Additional file 17 — The percentages of PtLRR-RLK genes from each subfamily being induced (A) or suppressed (B) for each treatment. [file 1471-2164-14-318-S17.doc]

Up-regulated PtLRR-RLK genes in different treatments,%: percentages of induced genes in each subfamily; FC: the average fold change of induced genes for each treatment.

| SG | GSE12152 shoot organogenesis | | | | | | | | GSE16783 wounding | | | | GSE13109 hypoxia | | | |
| --- | --- | --- | --- | --- | --- | --- | --- | --- | --- | --- | --- | --- | --- | --- | --- | --- |
|  | callus induce3d | FC | callus induce 15d | FC | shoot induce3d | FC | shoot induce 8d | FC | L5_1w | FC | L1_1w | FC | Leaf | FC | Rt | FC |
| I-a(9/16) |  |  |  |  |  |  |  |  | 22% | 9.4 | 33% | 2.5 | 56% | 3.0 |  |  |
| I-b(10/17) |  |  |  |  |  |  |  |  | 50% | 3.1 | 20% | 3.1 | 40% | 2.8 |  |  |
| II(20/29) | 5% | 2.2 | 5% | 2.1 | 5% | 2.3 | 5% | 2.2 | 45% | 5.0 |  |  | 40% | 3.4 |  |  |
| III(26/29) |  |  |  |  |  |  |  |  | 42% | 4.8 | 8% | 3.6 | 27% | 5.2 | 4% | 2.7 |
| IV(18/20) |  |  |  |  |  |  |  |  | 44% | 5.2 | 6% | 2.6 | 28% | 4.4 |  |  |
| V(9/11) |  |  |  |  |  |  |  |  | 56% | 8.0 | 11% | 2.1 | 22% | 5.9 | 11% | 3.1 |
| VI(14/16) |  |  |  |  |  |  |  |  | 36% | 5.0 | 7% | 3.0 | 14% | 2.2 |  |  |
| VII-a(6/6) | 17% | 2.2 |  |  | 17% | 2.6 |  |  |  |  | 33% | 3.2 | 83% | 6.0 |  |  |
| VII-b(17/30) |  |  |  |  |  |  |  |  | 6% | 6.9 | 18% | 2.8 | 59% | 4.6 | 6% | 2.0 |
| VIII(13/17) |  |  | 8% | 2.0 |  |  |  |  | 31% | 3.9 | 31% | 3.0 | 54% | 2.8 |  |  |
| IX(9/12) |  |  |  |  |  |  |  |  | 67% | 8.8 |  |  | 67% | 3.7 |  |  |
| X(21/29) |  |  |  |  |  |  |  |  | 14% | 2.6 | 14% | 2.7 | 52% | 3.1 | 10% | 3.1 |
| XI-a(23/28) | 4% | 2.6 | 4% | 2.6 | 9% | 2.6 | 4% | 2.7 | 26% | 3.8 | 9% | 3.7 | 26% | 3.3 |  |  |
| XI-b(18/20) |  |  |  |  |  |  | 6% | 2.2 | 61% | 8.5 | 6% | 2.7 | 33% | 4.6 | 6% | 3.0 |
| XI-c(7/7) |  |  |  |  |  |  |  |  | 14% | 3.1 |  |  | 57% | 2.6 |  |  |
| XII-a(18/23) | 11% | 2.3 |  |  | 6% | 2.1 | 6% | 2.1 | 22% | 3.8 | 22% | 5.6 | 61% | 4.1 | 6% | 2.2 |
| XII-b(16/19) | 6% | 2.2 | 13% | 2.7 | 13% | 2.7 | 19% | 2.7 | 13% | 8.0 | 6% | 3.8 | 56% | 4.6 | 6% | 2.7 |
| XIII(19/34) | 5% | 2.1 |  |  |  |  |  |  | 16% | 2.2 |  |  | 37% | 3.0 | 11% | 2.5 |
| XIV(7/10) | 14% | 2.0 |  |  | 14% | 2.1 |  |  | 14% | 4.8 | 14% | 2.3 | 29% | 3.1 | 14% | 2.1 |
| SG | GSE14893 N- | | | | | | GSE21480 Win&sum | | GSE16785 wounding | | | | GSE16773 methyl jasmonate1 | | | |
|  | L2_4w | FC | L5_4w | FC | L5_8w | FC | Pt_win | FC | L5_90h | FC | Rt_90h | FC | methyl 48h | FC |  |  |
| I-a(9/16) | 33% | 2.2 |  |  | 11% | 4.2 |  |  | 22% | 2.6 | 22% | 3.4 |  |  |  |  |
| I-b(10/17) |  |  | 10% | 5.3 |  |  |  |  | 10% | 4.3 | 20% | 3.1 |  |  |  |  |
| II(20/29) | 20% | 5.8 |  |  | 10% | 2.4 | 5% | 5.5 | 25% | 4.3 | 5% | 4.5 |  |  |  |  |
| III(26/29) | 12% | 5.4 | 15% | 4.7 | 8% | 5.5 | 19% | 5.7 | 50% | 3.1 | 12% | 3.8 | 27% | 3.7 |  |  |
| IV(18/20) | 17% | 2.6 | 11% | 5.1 | 6% | 2.0 |  |  | 56% | 4.8 | 6% | 3.3 | 11% | 2.6 |  |  |
| V(9/11) | 33% | 3.1 | 22% | 5.9 |  |  |  |  | 78% | 4.8 |  |  | 11% | 5.5 |  |  |
| VI(14/16) | 21% | 2.4 | 7% | 2.8 |  |  |  |  | 36% | 2.9 | 29% | 2.6 | 7% | 15.1 |  |  |
| VII-a(6/6) | 17% | 3.6 |  |  |  |  |  |  | 50% | 4.1 | 50% | 3.6 | 33% | 2.8 |  |  |
| VII-b(17/30) | 24% | 3.8 | 18% | 4.1 | 12% | 4.6 | 12% | 2.2 | 29% | 3.3 | 12% | 4.4 | 29% | 4.4 |  |  |
| VIII(13/17) | 15% | 10.1 | 15% | 2.7 | 38% | 2.0 | 8% | 4.4 | 38% | 3.9 | 62% | 5.9 | 23% | 3.5 |  |  |
| IX(9/12) | 11% | 2.2 |  |  |  |  | 33% | 2.5 | 44% | 4.4 | 22% | 2.7 |  |  |  |  |
| X(21/29) | 14% | 3.5 | 19% | 3.1 | 10% | 2.9 | 5% | 4.1 | 24% | 3.0 | 29% | 2.7 | 10% | 3.9 |  |  |
| XI-a(23/28) | 22% | 3.6 | 4% | 2.2 | 9% | 3.0 | 13% | 3.8 | 35% | 3.6 | 35% | 3.7 | 17% | 2.5 |  |  |
| XI-b(18/20) | 33% | 2.3 | 6% | 2.5 | 6% | 3.3 |  |  | 39% | 4.9 |  |  | 17% | 2.7 |  |  |
| XI-c(7/7) |  |  | 14% | 2.8 | 43% | 3.0 |  |  | 43% | 2.5 | 29% | 3.4 | 29% | 2.6 |  |  |
| XII-a(18/23) | 39% | 2.7 | 6% | 7.6 | 11% | 3.5 | 17% | 15.2 | 11% | 4.0 | 28% | 4.0 | 6% | 8.8 |  |  |
| XII-b(16/19) | 50% | 3.8 | 25% | 5.1 | 19% | 3.2 | 13% | 11.8 | 38% | 3.5 | 25% | 6.4 | 19% | 2.6 |  |  |
| XIII(19/34) | 26% | 2.8 | 11% | 3.0 | 11% | 2.9 | 16% | 5.4 | 47% | 2.6 | 26% | 6.4 | 5% | 2.2 |  |  |
| XIV(7/10) |  |  | 14% | 2.1 | 14% | 3.8 | 29% | 5.0 | 29% | 4.1 | 14% | 2.3 |  |  |  |  |

Down-regulated PtLRR-RLK genes in different treatments, %: percentages of repressed genes in each subfamily; FC: the average fold change of repressed genes for each treatment.

| SG | GSE12152 shoot organogenesis | | | | | | | | GSE16783 wounding | | | | GSE13109 hypoxia | | | |
| --- | --- | --- | --- | --- | --- | --- | --- | --- | --- | --- | --- | --- | --- | --- | --- | --- |
|  | callus induce3d | FC | callus induce15d | FC | shoot induce3d | FC | shoot induce15d | FC | L5_1w | FC | L1_1w | FC | Leaf | FC | Rt | FC |
| I-a(9/16) |  |  |  |  |  |  |  |  | 44% | 0.1 | 22% | 0.4 | 11% | 0.2 | 33% | 0.4 |
| I-b(10/17) |  |  | 20% | 0.5 | 10% | 0.5 |  |  |  |  | 20% | 0.4 |  |  | 10% | 0.2 |
| II(20/29) |  |  | 5% | 0.4 | 10% | 0.5 |  |  | 10% | 0.2 | 40% | 0.3 |  |  | 15% | 0.4 |
| III(26/29) |  |  | 4% | 0.3 | 4% | 0.4 |  |  | 19% | 0.3 | 19% | 0.3 | 4% | 0.1 | 27% | 0.4 |
| IV(18/20) |  |  |  |  | 6% | 0.5 |  |  | 6% | 0.1 | 22% | 0.4 | 6% | 0.3 | 39% | 0.4 |
| V(9/11) |  |  |  |  |  |  |  |  |  |  | 33% | 0.4 |  |  |  |  |
| VI(14/16) | 21% | 0.5 | 14% | 0.4 | 7% | 0.5 | 7% | 0.5 | 14% | 0.2 | 7% | 0.4 | 14% | 0.5 | 14% | 0.3 |
| VII-a(6/6) |  |  |  |  |  |  |  |  | 100% | 0.3 | 33% | 0.4 |  |  | 17% | 0.4 |
| VII-b(17/30) | 6% | 0.5 | 6% | 0.4 | 6% | 0.3 | 6% | 0.4 | 59% | 0.3 | 47% | 0.3 | 6% | 0.5 | 29% | 0.2 |
| VIII(13/17) | 15% | 0.5 | 31% | 0.4 | 15% | 0.4 | 31% | 0.5 | 38% | 0.3 | 8% | 0.4 | 8% | 0.3 | 46% | 0.4 |
| IX(9/12) |  |  |  |  | 11% | 0.5 |  |  | 11% | 0.5 |  |  |  |  | 67% | 0.4 |
| X(21/29) |  |  |  |  |  |  |  |  | 38% | 0.2 | 19% | 0.3 |  |  | 14% | 0.4 |
| XI-a(23/28) | 4% | 0.3 | 13% | 0.4 | 4% | 0.2 | 4% | 0.2 | 22% | 0.2 | 35% | 0.3 | 4% | 0.5 | 70% | 0.4 |
| XI-b(18/20) | 17% | 0.4 | 11% | 0.3 | 17% | 0.4 | 11% | 0.4 |  |  | 22% | 0.3 | 11% | 0.3 | 28% | 0.3 |
| XI-c(7/7) |  |  | 14% | 0.5 | 14% | 0.3 |  |  | 43% | 0.1 | 14% | 0.1 |  |  | 57% | 0.3 |
| XII-a(18/23) | 6% | 0.5 | 6% | 0.5 |  |  |  |  | 28% | 0.1 | 22% | 0.3 |  |  | 17% | 0.3 |
| XII-b(16/19) | 6% | 0.4 | 6% | 0.4 | 6% | 0.4 | 6% | 0.4 | 44% | 0.2 | 25% | 0.2 | 6% | 0.3 | 19% | 0.4 |
| XIII(19/34) | 5% | 0.4 | 5% | 0.2 | 5% | 0.3 | 5% | 0.3 | 21% | 0.3 | 11% | 0.5 |  |  | 16% | 0.4 |
| XIV(7/10) |  |  |  |  |  |  |  |  | 57% | 0.1 | 29% | 0.2 |  |  |  |  |
| SG | GSE14893 N- | | | | | | GSE21480 Win&sum | | GSE16785 wounding | | | | GSE16773 methyl jasmonate1 | | | |
|  | L2_4w | FC | L5_4w | FC | L5_8w | FC | Pt_win | FC | L5_90h | FC | Rt_90h | FC | methyl 48h | FC |  |  |
| I-a(9/16) | 22% | 0.3 | 56% | 0.4 | 11% | 0.3 | 89% | 0.2 |  |  |  |  | 22% | 0.4 |  |  |
| I-b(10/17) | 10% | 0.2 |  |  | 10% | 0.4 | 60% | 0.3 | 30% | 0.4 |  |  | 10% | 0.4 |  |  |
| II(20/29) | 5% | 0.2 | 20% | 0.3 | 25% | 0.2 | 70% | 0.1 | 10% | 0.4 | 5% | 0.3 | 10% | 0.4 |  |  |
| III(26/29) | 8% | 0.2 | 23% | 0.3 | 46% | 0.3 | 50% | 0.1 | 4% | 0.4 | 15% | 0.3 | 8% | 0.3 |  |  |
| IV(18/20) |  |  | 22% | 0.3 | 50% | 0.3 | 100% | 0.1 | 6% | 0.2 | 11% | 0.4 | 17% | 0.3 |  |  |
| V(9/11) |  |  | 33% | 0.4 | 33% | 0.3 | 56% | 0.1 | 11% | 0.3 | 33% | 0.3 | 22% | 0.3 |  |  |
| VI(14/16) | 7% | 0.2 | 29% | 0.3 | 14% | 0.2 | 86% | 0.2 |  |  |  |  | 7% | 0.2 |  |  |
| VII-a(6/6) |  |  | 17% | 0.2 |  |  | 33% | 0.2 | 33% | 0.3 |  |  | 17% | 0.2 |  |  |
| VII-b(17/30) | 6% | 0.1 | 29% | 0.4 | 29% | 0.3 | 41% | 0.2 | 12% | 0.3 | 12% | 0.4 | 24% | 0.3 |  |  |
| VIII(13/17) | 15% | 0.5 | 23% | 0.4 | 15% | 0.2 | 31% | 0.1 |  |  |  |  | 23% | 0.3 |  |  |
| IX(9/12) |  |  | 11% | 0.2 | 44% | 0.3 | 56% | 0.1 |  |  | 11% | 0.5 | 11% | 0.3 |  |  |
| X(21/29) |  |  | 14% | 0.2 | 14% | 0.3 | 38% | 0.2 | 5% | 0.4 | 5% | 0.2 | 29% | 0.4 |  |  |
| XI-a(23/28) | 4% | 0.2 | 17% | 0.3 | 22% | 0.4 | 65% | 0.1 | 0% |  |  |  | 9% | 0.3 |  |  |
| XI-b(18/20) |  |  | 28% | 0.3 | 67% | 0.3 | 56% | 0.2 | 6% | 0.3 | 17% | 0.2 | 28% | 0.2 |  |  |
| XI-c(7/7) | 14% | 0.4 | 29% | 0.2 | 14% | 0.5 | 43% | 0.1 |  |  |  |  | 43% | 0.3 |  |  |
| XII-a(18/23) | 6% | 0.5 | 17% | 0.3 | 39% | 0.3 | 28% | 0.3 | 22% | 0.2 | 22% | 0.3 | 33% | 0.4 |  |  |
| XII-b(16/19) | 6% | 0.4 | 19% | 0.3 | 19% | 0.3 | 38% | 0.3 | 19% | 0.3 | 13% | 0.5 | 38% | 0.2 |  |  |
| XIII(19/34) | 5% | 0.4 | 16% | 0.4 | 16% | 0.3 | 16% | 0.2 |  |  |  |  | 21% | 0.4 |  |  |
| XIV(7/10) | 14% | 0.4 | 29% | 0.3 | 29% | 0.4 |  |  | 14% | 0.1 |  |  |  |  |  |  |
