# Supplementary material for: Genome-wide identification, characterization and expression analysis of populus leucine-rich repeat receptor-like protein kinase genes
Source: BMC Genomics. 2013 May 10;14:318. doi: 10.1186/1471-2164-14-318 (PMC3682895; doi:10.1186/1471-2164-14-318)
Supplement: Additional file 18 — Information on the AGI code, gene full name and abbreviation for each AtLRR-RLK gene with defined functions presented in this work. [file 1471-2164-14-318-S18.pdf]

LRR-XI	BAM1	AT5G65700	Barely any meristem1
LRR-XI	BAM2	AT3G49670	Barely any meristem2
LRR-XI	BAM3	AT4G20270	Barely any meristem3
LRR-Xa	BIR1	AT5G48380	BAK1-interacting receptor-like kinase like1
LRR-Xb	BRI1	AT4G39400	Brassinosteroid insensitive1
LRR-Xb	BRL1	AT1G55610	BAK1-interacting receptor-like kinase like1
LRR-Xb	BRL2	AT2G01950	BAK1-interacting receptor-like kinase like2
LRR-Xb	BRL3	AT3G13380	BAK1-interacting receptor-like kinase like3
LRR-XV	CLI1	AT3G02130	CLV3 peptide insensitive 1
LRR-XI	CLV1	AT1G75820	CLAVATA 1
LRR-I	CRLK1	AT5G54590	Calcium/calmodulin-regulated receptor-like kinase 1
LRR-I	CRLK2	AT5G15730	Calcium/calmodulin-regulated receptor-like kinase 2
LRR-XI	EDA23	AT5G44700	Embryo sac development arrest23
LRR-XII	EFR	AT5G20480	EF-TU receptor
LRR-Xb	EMS1	AT5G07280	Excess microsporocytes1
LRR-XIIIb	ERECTA	AT2G26330	ERECTA
LRR-XIIIb	ERL1	AT5G62230	ERECTA-LIKE 1
LRR-XIIIb	ERL2	AT5G07180	ERECTA-LIKE 2
LRR-XIIIa	FEI1	AT1G31420	named for the Chinese word for fat
LRR-XIIIa	FEI2	AT2G35620	named for the Chinese word for fat
LRR-XII	FLS2	AT5G46330	Flagellin-sensitive-2
LRR-I	FRK1	AT2G19190	Flg22-induced receptor-like kinase 1
LRR-XI	GSO1	AT4G20140	GASSHO1
LRR-XI	HAE	AT4G28490	HAESA
LRR-XI	HAIKU2	AT3G19700	
LRR-III	IMK2	AT3G51740	Inflorescence meristem receptor-like kinase2
LRR-III	IMK3	AT3G56100	Inflorescence meristem receptor-like kinase3
LRR-I	IOS1	AT1G51800	Impaired oomycete susceptibility
LRR-III	LRR1	AT5G16590	Leucine-rich repeat 1
LRR-I	MEE39	AT3G46330	Maternal effect embryo arrest 39
LRR-VII	MEE62	AT5G45800	Maternal effect embryo arrest 62
LRR-XIV	MOL1	AT5G51350	More lateral growth1
LRR-VI-2	MRH1	AT4G18640	Morphogenesis of root hair 1
LRR-II	NIK1	AT5G16000	NSP-interacting kinase 1
LRR-II	NIK2	AT3G25560	NSP-interacting kinase 2
LRR-II	NIK3	AT1G60800	NSP-interacting kinase 3
LRR-XI	PEPR1	AT1G73080	PEP1 receptor 1
LRR-XI	PEPR2	AT1G17750	PEP1 receptor 2
LRR-III	PRK2A	AT2G07040	Pollen receptor like kinase 2
LRR-Xb	PSKR1	AT2G02220	Phytosulfokin receptor 1
LRR-Xb	PSKR2	AT5G53890	Phytosulfokin receptor 2
LRR-XI	PXY	AT5G61480	Phloem intercalated with xylem
LRR-III	RKL1	AT1G48480	Rreceptor-like kinase 1
LRR-XI	RLK7	AT1G09970	LRR XI-23 Rreceptor-like kinase 7
LRR-III	RLK902	AT3G17840	Rreceptor-like kinase 902
LRR-XV	RPK1	AT1G69270	Rreceptor-like protein kinese 1
LRR-III	RUL1	AT5G05160	Reduced in lateral growth1
LRR-II	SARK	AT4G30520	Senescence-associated Rreceptor-like kinase
LRR-V	SCM	AT1G11130	Scrambled
LRR-II	SERK1	AT1G71830	Somatic embryogenesis receptor-like kinase1
LRR-II	SERK2	AT1G34210	Somatic embryogenesis receptor-like kinase2
LRR-II	SERK3	AT4G33430	Somatic embryogenesis receptor-like kinase3
LRR-II	SERK4	AT2G13790	Somatic embryogenesis receptor-like kinase4

LRR-II	SERK5	AT2G13800	Somatic embryogenesis receptor-like kinase5
LRR-V	SRF1	AT2G20850	Strubbelig-receptor family 1
LRR-V	SRF2	AT5G06820	Strubbelig-receptor family 2
LRR-V	SRF3	AT4G03390	Strubbelig-receptor family 3
LRR-V	SRF4	AT3G13065	Strubbelig-receptor family 4
LRR-V	SRF5	AT1G78980	Strubbelig-receptor family 5
LRR-V	SRF6	AT1G53730	Strubbelig-receptor family 6
LRR-V	SRF7	AT3G14350	Strubbelig-receptor family 7
LRR-V	SRF8	AT4G22130	Strubbelig-receptor family 8
LRR-III	TMKL1	AT3G24660	Transmember kinese-like 1
LRR-XI	XIP1	AT5G49660	Xylem intermixed with phloem 1
